# Revealing Dynamic Ion Transport in Tailorable Carbon Nano‐Skyscraper Electrodes

**DOI:** 10.1002/advs.202503749

**Published:** 2025-06-05

**Authors:** Jiye Li, Xiaoyang Zheng, Luting Zhu, Yihang Yao, Sisi Yan, Lang Wang, Jing He, Chen Zhao, Ziqian Zhou, Liaoyong Wen

**Affiliations:** ^1^ School of Materials Science and Engineering Zhejiang University Hangzhou 310027 China; ^2^ Research Center for Industries of the Future (RCIF) Zhejiang Key Laboratory of 3D Micro/Nano Fabrication and Characterization School of Engineering Westlake University Hangzhou 310030 China; ^3^ Institute of Engineering Innovation Graduate School of Engineering The University of Tokyo Tokyo 113‐8656 Japan; ^4^ Westlake Institute for Optoelectronics Fuyang Hangzhou 311421 China

**Keywords:** 3D carbon nanotubes, anodic aluminum oxide, electrochemical energy storage, ion transport, low‐curvature electrodes, nanoporous electrodes

## Abstract

Optimizing ion transport dynamics in nanoporous electrodes is crucial for advancing electrochemical energy storage and conversion technologies. However, rapid charge relaxation and architectural complexity in conventional electrodes impede a comprehensive understanding of ionic behavior. Here, convoluted ion migration routes are decoupled into two distinct pathways by precisely engineering the density and spatial arrangement of 3D carbon‐interconnected nanoporous architectures. The findings reveal that ions exhibit time‐optimized transport, prioritizing pathways that minimize temporal resistance over shorter spatial distances. This behavior, enabled by rational electrode design, enhances the performance of quasi‐ideal (low‐curvature) electrodes by 20% at ultrahigh scan rates of 1 0000 mV s^−1^. Through finite element simulations and experimental validation, it is further demonstrated that uniformly distributed nanoporous configurations outperform localized and gradient designs in charging dynamics. These insights provide a framework for designing high‐efficiency nanoporous electrodes, with significant implications for next‐generation electrochemical devices.

## Graphical abstract

A 3D carbon nanotube electrode reveals how ions and electrons prioritize rapid transport over the shortest paths during charging. By creating a uniformly interconnected porous network, researchers improved electrode efficiency, surpassing traditional localized and gradient designs. This optimized architecture reduces transport resistance and can guide the development of future high‐performance devices.

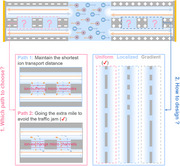

## 1 Introduction

The global transition toward sustainable energy systems urgently demands advancements in electrochemical energy conversion and storage technologies, such as batteries and supercapacitors.^[^ ^1^, ^2^ ^]^ These devices rely on two coupled processes: rapid electron transfer through external circuits and slower, more complex ion transport within nanoporous electrodes.^[^ ^3^, ^4^, ^5^, ^6^ ^]^ While electron kinetics are well‐optimized, ion mobility remains a critical bottleneck, governed by intricate interactions between ions and the tortuous, heterogeneous nanostructures of electrodes. Despite progress in material design and electrochemistry, the persistent disconnect between idealized models and real‐world electrode architectures limits the full understanding and optimization of ion dynamics.^[^ ^4^, ^7^, ^8^, ^9^, ^10^, ^11^ ^]^

Central to ion transport theory is Einstein's formulation of Brownian motion ($x^{2}\;\left(t\right)=2Dt$), which relates mean square displacement to diffusion coefficient (*D*) and time (*t*).^[^ ^12^ ^]^ Extensions of this framework incorporate structural parameters such as tortuosity and porosity to describe ion movement in confined geometries.^[^ ^7^, ^13^, ^14^ ^]^ These insights have driven innovations in electrode engineering, including aligned graphite,^[^ ^15^ ^]^ liquid‐crystalline MXenes,^[^ ^16^ ^]^ and nanotextured ruthenium nitride,^[^ ^17^ ^]^ which reduce tortuosity by streamlining ion pathways. Complementary strategies, such as grooved electrodes^[^ ^8^ ^]^ and porous current collectors,^[^ ^18^ ^]^ further shorten transport distances. However, these efforts predominantly focus on geometric optimization, neglecting the impact of inherent structural heterogeneities—local pore size variations, defects, and irregular connectivity—that induce spatially nonuniform ion transport. Such heterogeneity, pervasive in practical electrodes, may create kinetic imbalances during charging, yet its influence remains poorly quantified.^[^ ^19^, ^20^, ^21^, ^22^, ^23^, ^24^ ^]^

Experimental probes like nuclear magnetic resonance,^[^ ^9^, ^23^, ^24^, ^25^, ^26^ ^]^ electrochemical quartz crystal microbalance,^[^ ^27^ ^]^ in situ infrared spectroscopy,^[^ ^28^ ^]^ and X‐ray scattering^[^ ^22^ ^]^ have provided snapshots of ion behavior, but their limited spatiotemporal resolution and protracted data acquisition hinder dynamic characterization.^[^ ^22^, ^29^ ^]^ Molecular dynamics (MD) simulations, while powerful for analyzing nanoscale ion‐pore interactions,^[^ ^11^, ^30^, ^31^, ^32^, ^33^, ^34^, ^35^, ^36^, ^37^ ^]^ face computational constraints that restrict simulations to simplistic, single‐pore models or systems smaller than 100 nm.^[^ ^20^, ^21^ ^]^ These idealized representations fail to capture the multiscale complexity of interconnected pore networks in real electrodes, leaving a critical gap in understanding how structural irregularities at practical scales govern collective ion dynamics.

**Figure 1:**
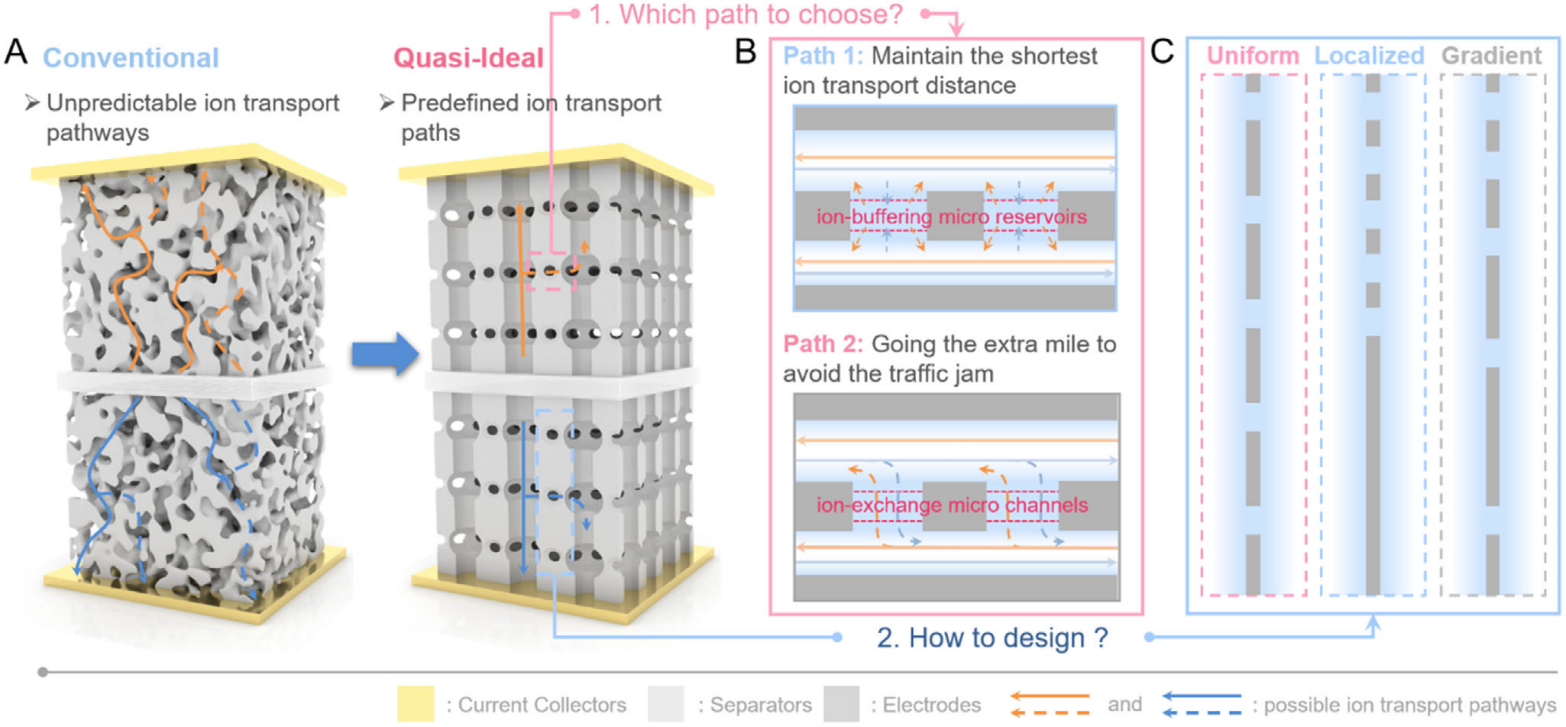
The design concept of carbon nano‐skyscraper electrodes. A) Schematic diagram of the assembly structure of a supercapacitor. Left: A representative conventional electrode. Right: The quasi‐ideal electrode proposed in this work. The microstructure of traditional electrodes is highly complex, leading to unpredictable ion transport pathways. Our work can predefine the ion transport pathways by controlling the structure of the quasi‐ideal electrode. B) Two possible ion transport pathways in nanoporous electrodes. C) Three possible candidate schemes for the optimal nanoporous design: uniform, localized, and gradient designs.

Here, we address this challenge using 3D carbon‐coated anodized aluminum oxide (3D C‐AAO) electrodes as a tunable model system. The 3D C‐AAO architecture features vertically aligned “nano‐skyscraper” pores, with transverse channels perforating their walls (**Figure** **1**A). By systematically varying the density and spatial distribution of these transverse pores, we decouple the effects of transport path geometry (direct vs. tortuous) and structural heterogeneity on ion dynamics. We demonstrate that ion migration during charging prioritizes minimal‐time paths over shorter spatial distances (Figure 1B) and that uniformly distributed transverse pores mitigate ion concentration gradients arising from inherent pore size variations (Figure 1C). Supported by finite element simulations, we confirm that uniformly distributed transverse pores lead to the most significant improvement in charging dynamics, outperforming both localized and gradient configurations. This work establishes a design principle for reconciling geometric optimization with heterogeneity control in nanoporous electrodes, advancing the rational engineering of high‐performance energy storage systems.

## 2 Results and Discussion

### 2.1 Fabrication of 3D C‐AAO Electrodes

**Figure 2:**
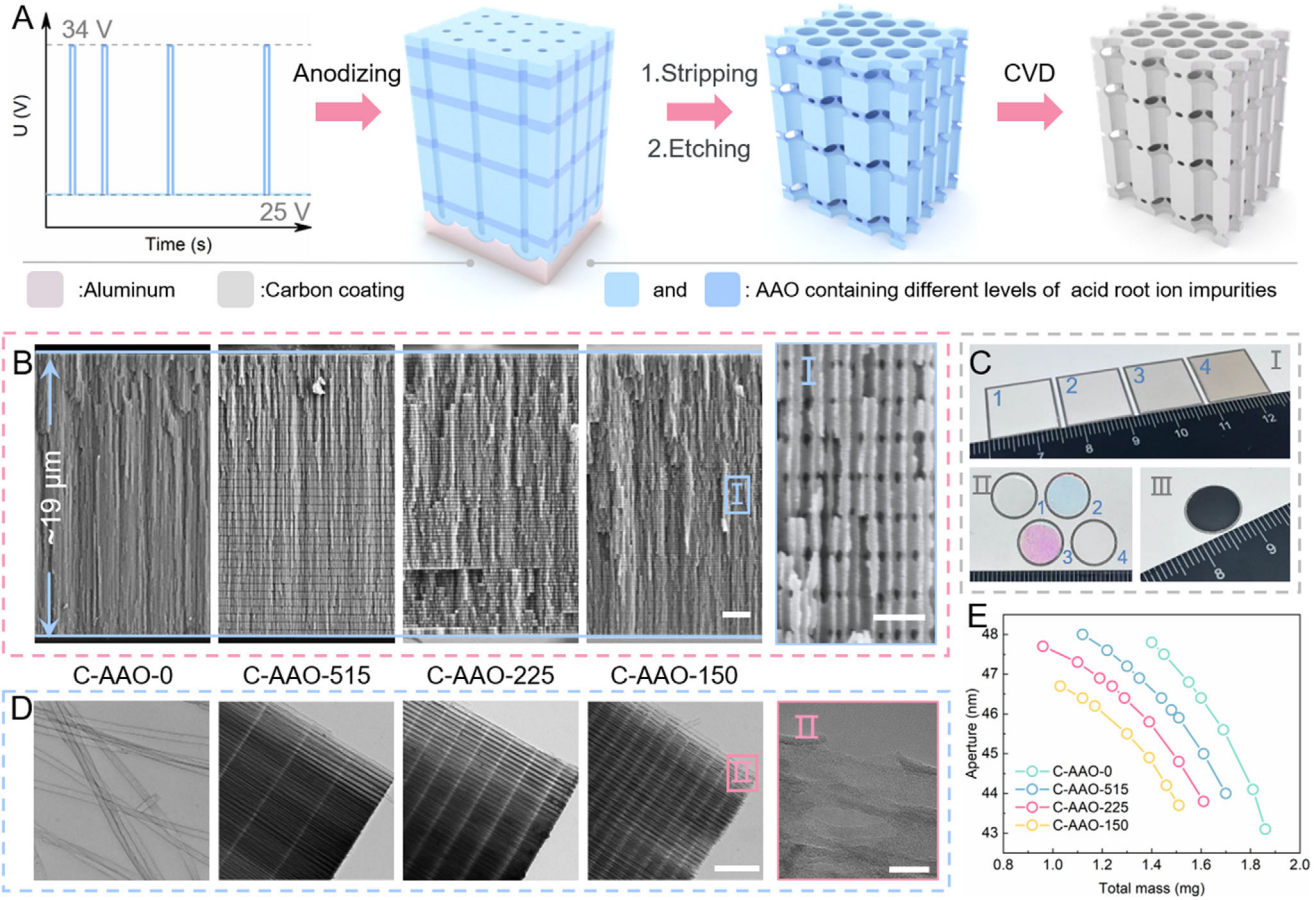
Preparation process and structural characterization of 3D C‐AAO electrodes. A) Schematic diagram illustrating the preparation process for the 3D C‐AAO electrodes. B) Cross‐sectional SEM images of C‐AAO‐0, C‐AAO‐515, C‐AAO‐225, and C‐AAO‐150. (I) Enlarged cross‐sectional SEM image. C) Optical photographs of samples at various stages: (I) Peeled 3D AAO template from the aluminum substrate. 1, 2, 3, and 4 are the precursors of C‐AAO‐0, C‐AAO‐515, C‐AAO‐225, and C‐AAO‐150, respectively. Increasing the number of pulses leads to more acid ion impurities in the material, thereby reducing the transparency of the AAO. (II) 3D AAO templates after etching and heat treatment. The heat treatment process removes acid ion impurities, making the 3D AAO templates more transparent. (III) Representative 3D C‐AAO after CVD. D) TEM images of C‐AAO‐0, C‐AAO‐515, C‐AAO‐225, and C‐AAO‐150 after the removal of the 3D‐AAO template. (II) Enlarged TEM image. Note: The removal of the 3D AAO template was performed to observe the carbon film more clearly. All subsequent tests were conducted using 3D C‐AAO electrodes with the 3D AAO framework intact. E) Relationship between electrode mass and pore aperture of 3D C‐AAO templates. Scale bars: (B) 2 µm, 200 nm; (D) 500, 20 nm.

The process for preparing the 3D C‐AAO electrodes is depicted in **Figure** **2**A (a detailed synthesis process is outlined in the materials and methods procedures, Figure S1 and Table S1, Supporting Information). In brief, a pulse anodization technique was used to create the 3D AAO template, followed by thermal pretreatment (Figure S2, Supporting Information) and chemical vapor deposition (CVD) to produce robust 3D C‐AAO electrodes. The size and distribution of the transversal nanopores in the 3D C‐AAO can be precisely controlled (Figures S3 and S4, Supporting Information). For example, four variants—C‐AAO‐0, C‐AAO‐515, C‐AAO‐225, and C‐AAO‐150—were synthesized with transversal nanopore spacings of 0, 515, 225, and 150 nm, respectively. Cross‐sectional SEM images confirmed uniform pore distributions (Figure 2B).

Representative optical images in Figure 2C (Region I) highlight key stages of sample preparation. The high‐voltage pulses introduced additional acid‐root ion impurities into the 3D AAO templates, giving the templates deeper colors. Furthermore, due to their periodic 3D structures, 3D AAO templates exhibit properties similar to those of distributed Bragg reflectors or 1D photonic crystals.^[^ ^38^, ^39^ ^]^ Changes in the periodic intervals cause color variations in the samples (Figure 2C, Region II, and Figure S5, Supporting Information). After CVD, robust and self‐supporting 3D C‐AAO electrodes with carbon coatings of consistent defect levels were obtained, making them ideal for direct electrochemical testing and analysis (Figure 2C, Region III, and Figures S6 and S7, Supporting Information).

TEM images in Figure 2D and SEM images in Figure S8 (Supporting Information) show the carbon coating left after removing the 3D AAO template. The formation of transversal nanopores allows isolated carbon nanotubes to connect seamlessly through C─C covalent bonds, maintaining structural integrity and emphasizing the role of transversal nanopores as interconnected channels and electron pathways.^[^ ^40^, ^41^ ^]^

We used ImageJ software to analyze all 3D C‐AAO SEM images and generate nanopore size distribution graphs (Figures S9–S16, Supporting Information).^[^ ^42^ ^]^ As shown in Figure 2E, the average nanopore size of the electrode increases as its mass decreases, primarily due to the reduction in the 3D AAO template mass. All 3D C‐AAO electrodes exhibit similar curve trends, but the formation of transversal nanopores also reduces the 3D AAO template mass, shifting the curves of electrodes with more transversal nanopores leftward along the X‐axis. These transversal nanopores form due to the preferential dissolution of the AAO template containing high sulfate ion impurities. Insufficient etching time leads to the formation of bamboo‐like pores rather than fully interconnected transversal nanopores. In our experiments, transversal nanopores formed when the pore size exceeded 45 nm (Figures S17–S19 and Table S2, Supporting Information).

### 2.2 Ion Dynamics in 3D C‐AAO Electrodes with Different Densities of Transversal Nanopores

**Figure 3:**
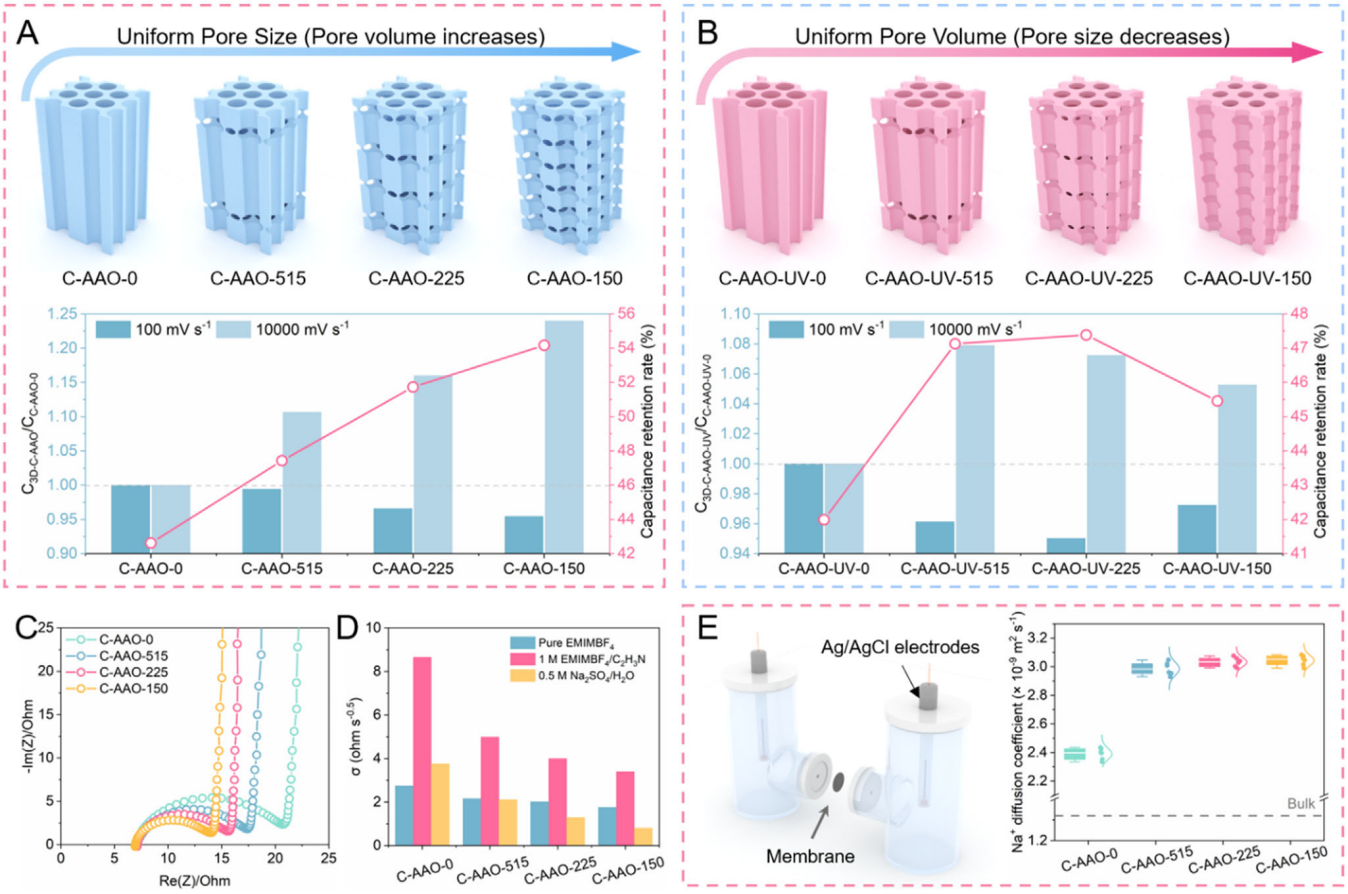
Effect of transversal nanopore structures on ion transport. A) Top: Schematic diagram illustrating the structure of 3D C‐AAO electrodes with uniform pore size. Bottom: Normalized capacitance C_C‐AAO_/C_C‐AAO‐0_ and capacitance retention of the corresponding 3D C‐AAO electrodes, measured in pure ionic liquid electrolyte. Unless specified otherwise, all tests were conducted on assembled symmetric supercapacitors. B) Top: Schematic diagram illustrating the structure of 3D C‐AAO electrodes with uniform pore volume (C‐AAO‐UV). Bottom: Normalized capacitance C_C‐AAO‐UV_/C_C‐AAO‐UV‐0_ and capacitance retention of the corresponding C‐AAO‐UV electrodes, measured in pure ionic liquid electrolyte. C) Nyquist plot of the 3D C‐AAO electrodes in pure ionic liquid electrolyte. D) Warburg coefficient (σ) of the 3D C‐AAO electrodes in different electrolyte systems. E) Left: Schematic of an electrolytic cell used to test *I*–*V* curves. Right: Na^+^ diffusion coefficient of 3D C‐AAO electrodes, measured in 0.5 m Na_2_SO_4_ aqueous solution. The gray dashed lines in the figures represent the Na^+^ diffusion coefficients in an aqueous solution without the 3D C‐AAO membranes.

Through a controlled electrode preparation and screening process (Figure S20, Supporting Information), we fabricated 3D C‐AAO electrodes with varying densities of transversal nanopores to investigate the relationship between structure and ion dynamics. Before analysis, we confirmed the excellent electrochemical and mechanical stability of the C‐AAO electrodes (Figure S21, Supporting Information). The capacitance and capacitance retention of the 3D C‐AAO with identical straight nanopore sizes (unless otherwise specified, all subsequent comparisons are based on samples with the same nanopore size) were evaluated using cyclic voltammetry (CV). As shown in **Figure** **3**A, at a low scan rate of 100 mV s^−1^, the specific capacitance of the electrodes decreases with increasing density of transversal nanopores, corresponding to a reduction in surface area (Figures S22,S23 and Table S3, Supporting Information). Notably, C‐AAO‐150 exhibits the lowest specific capacitance, reaching only 95.4% of that of C‐AAO‐0.

As the scan rate increases, performance shifts from being primarily influenced by structure and composition to being governed by ion dynamics. At a scan rate of 1 0000 mV s^−1^, the C‐AAO‐150 electrode achieves the highest specific capacitance of 2.32 mF cm^−2^, which is ≈1.24 times greater than that of C‐AAO‐0 (Figure S23, Supporting Information). Capacitance retention improves with a higher density of transversal nanopores, with C‐AAO‐150 showing a retention rate of 54.2%, outperforming C‐AAO‐225, C‐AAO‐515, and C‐AAO‐0 with retention rates of 51.7%, 47.4%, and 42.6%, respectively (Figure 3A). The performance of 3D C‐AAO electrodes in aqueous electrolytes is consistent with their behavior in ionic liquids, suggesting the generality of the conclusions that the introduction of transversal nanopores enhances the charging dynamics of the electrode (Figure S24, Supporting Information).

However, the transversal nanopores increase the electrode's pore volume, which may also improve charging dynamics.^[^ ^3^, ^4^ ^]^ It remains uncertain whether these transversal nanopores function as “overpasses” (ion channels) during charging‐discharging cycles (Table S4, Supporting Information). To isolate the effect of pore volume on charging dynamics, we prepared 3D C‐AAO electrodes with uniform pore volumes (C‐AAO‐UV), designated C‐AAO‐UV‐0, C‐AAO‐UV‐515, C‐AAO‐UV‐225, and C‐AAO‐UV‐150. Increasing the density of the transversal pores reduces the overall pore size (both vertical and transversal) (Figure 3B; Figure S22, and Tables S3 and S4, Supporting Information). Thus, for C‐AAO‐UV‐150, the transversal pores are fully closed, resulting in a bamboo‐like structure.

The specific capacitances of C‐AAO‐UV electrodes initially decrease and then increase at a scan rate of 100 mV s^−1^, due to the competing effects of changes in the size of straight and transversal nanopores on the material's surface area (Figure 3B; Figure S25 and Table S4, Supporting Information). At a higher scan rate of 1 0000 mV s^−1^, the electrodes with transversal nanopores, specifically C‐AAO‐UV‐515 and C‐AAO‐UV‐225, outperform those with straight nanopores (C‐AAO‐UV‐0) and bamboo‐like pores (C‐AAO‐UV‐150) (Figures 3B; Figure S25, Supporting Information). This confirms again that ion exchange within the transversal nanopores facilitates the enhanced charging dynamics of the 3D C‐AAO electrodes.

Electrochemical impedance spectroscopy (EIS) provides valuable insights into the effects of transversal nanopores on ion dynamics. As shown in Figure 3C; Figure S26 (Supporting Information), all 3D C‐AAO electrodes exhibit consistent equivalent series resistance (ESR) across the tested electrolyte systems (7 Ω in pure 1‐Ethyl‐3‐methylimidazolium Tetrafluoroborate (Emim‐BF₄), 1 Ω in 1 m Emim‐BF₄/C_2_H_3_N, and 1 Ω in 0.5 m Na₂SO₄/H_2_O). This consistency highlights the stability of the testing system. The charge transfer resistance decreases as the density of transversal nanopores increases, which can be attributed to the formation of a conductive “electron transport network” through these nanopores, allowing for more efficient electron movement across the electrode. Among the samples, C‐AAO‐150 shows the steepest linear slope in the low‐frequency region, indicating the most favorable ion dynamics. This suggests that an increased density of transversal nanopores improves ion transport. To further quantify the effect of these nanopores on ion dynamics, we used the Warburg coefficient (σ) to measure diffusion resistance during ion transport (Figure 3D; Figure S26, Supporting Information). C‐AAO‐0 consistently exhibits the highest σ values across different electrolyte systems. However, with an increased density of transversal nanopores, the σ values decrease and eventually plateau. C‐AAO‐150 exhibits the lowest σ values (1.76‐, 3.40‐, and 0.81 Ω s^−0.5^, respectively), indicating that while transversal nanopores enhance ion transport, their benefits diminish with further increases in pore quantity.

To further solidify the relationship between transversal nanopores and ion transport, we assemble an electrochemical cell with 3D C‐AAO electrodes, measure ionic conductivity using *I*–*V* curves, and calculate the ionic diffusion coefficient via the Nernst‐Einstein equation (Figure 3E; Figure S27, Supporting Information). We find that the ionic diffusion coefficients for all 3D C‐AAO electrodes were higher than those in the bulk solution (without 3D C‐AAO electrodes), indicating that the introduction of 3D C‐AAO electrodes significantly enhances ion diffusion. This improvement can be attributed to the high surface charge and low friction of the carbon nanochannels.^[^ ^43^, ^44^ ^]^ Additionally, the ionic diffusion coefficient increases with the density of transversal nanopores until it reaches saturation (Figure 3E; Figure S27, Supporting Information), indicating that building transversal nanopores approximately every 500 nm delivers the most substantial and cost‐effective improvement in ion transport. Most importantly, our study challenges the conventional assumptions about ion transport in traditional nanoporous electrodes, which posit that ions and electrons within an electrode follow the shortest spatial route rather than paths of least resistance.^[^ ^4^, ^45^, ^46^ ^]^ Even in low‐curvature electrodes, the strategic configuration of transversal nanopores significantly enhances charging dynamics.

### 2.3 Ion Dynamics in 3D C‐AAO Electrodes with Different Spatial Distributions of Transversal Nanopores

**Figure 4:**
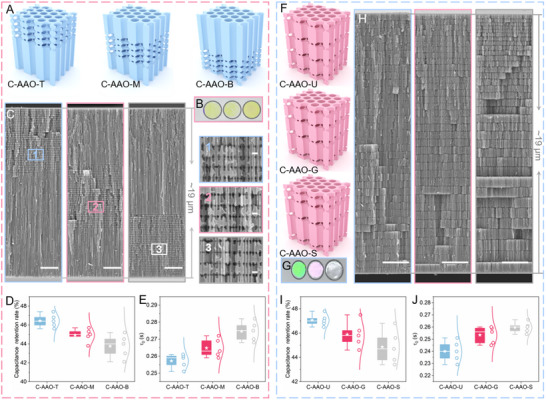
Effect of transversal nanopore distribution on ionic dynamics. A) Schematic diagram illustrating 3D C‐AAO electrodes with varying transversal pore spatial distributions: top (C‐AAO‐T), middle (C‐AAO‐M), and bottom (C‐AAO‐B). The top region corresponds to the area closest to the electrolyte. B) Optical image of the 3D AAO precursor of C‐AAO‐T, C‐AAO‐M, and C‐AAO‐B. C) SEM images of C‐AAO‐T, C‐AAO‐M, and C‐AAO‐B electrodes, Right: Enlarged SEM images of the corresponding areas 1, 2, and 3. D) Capacitance retention of the 3D C‐AAO electrodes at a scan rate of 10,000 mV s^−1^. E) Relaxation times (τ_0_) of the C‐AAO‐T, C‐AAO‐M, and C‐AAO‐B electrodes. F) Schematic diagram illustrating the structure of the 3D C‐AAO electrodes with transversal nanopores exhibiting uniform distribution (C‐AAO‐U), mild gradient (C‐AAO‐G), and steep gradient (C‐AAO‐S). G) Optical image of the 3D‐AAO precursors of C‐AAO‐U, C‐AAO‐G, and C‐AAO‐S. H) SEM images of the corresponding C‐AAO‐U, C‐AAO‐G, and C‐AAO‐S electrodes. I) Capacitance retention of the C‐AAO‐U, C‐AAO‐G, and C‐AAO‐S electrodes at a scan rate of 1 0000 mV s^−1^. J) τ_0_ of the C‐AAO‐U, C‐AAO‐G, and C‐AAO‐S electrodes. Scale bars: (C) 2 µm, 100 nm; (H) 2 µm.

To optimize structural design for enhanced performance, we investigated the effects of varying spatial distributions of transversal nanopores on charging dynamics. We fabricated 3D C‐AAO electrodes with consistent thickness, transversal pore density, and spacing but differing spatial distributions: top (C‐AAO‐T), middle (C‐AAO‐M), and bottom (C‐AAO‐B) (**Figure** **4**A). Optical and SEM images confirmed the successful fabrication of such structures (Figure 4B,C; Figure S28, Supporting Information). At low operating rates, these electrodes exhibited nearly identical specific capacitance and internal resistance, indicating that the distribution of transversal nanopores had minimal effect on surface properties and conductivity (Figure S29, Supporting Information). However, at a scan rate of 10,000 mV s^−1^, C‐AAO‐T showed capacitance retention of ≈46.4%, outperforming C‐AAO‐M (≈44.9%) and C‐AAO‐B (≈43.8%) (Figure 4D). This demonstrates the impact of the transversal nanopore distributions on charging dynamics, particularly when the nanopores are closer to the electrolyte‐electrode interface. This result is validated by EIS measurements, where C‐AAO‐T shows the shortest relaxation time (τ_0_) (≈0.257 s) compared to C‐AAO‐M (≈0.264 s) and C‐AAO‐B (≈0.274 s) (Figure 4E: Figure S29, Supporting Information).

Given that transversal nanopores near the electrolyte‐electrode interface demonstrated the most significant performance improvements, we assume that a top‐down gradient structural design may be optimal. To test this hypothesis, we prepared 3D C‐AAO electrodes with uniformly distributed transversal nanopores (C‐AAO‐U) and gradient distributions: C‐AAO‐G (mild gradient) and C‐AAO‐S (steep gradient) (Figure 4F). Optical and SEM images confirmed the differences in transversal nanopore distributions (Figure 4G,H; Figure S30, Supporting Information). In C‐AAO‐U, pore spacing is ≈515 nm, while C‐AAO‐G ranges from 380 nm at the top to 680 nm at the bottom, and C‐AAO‐S from 255 nm at the top to 800 nm at the bottom. As previously analyzed, the distribution of transversal pores does not affect low‐frequency performance (Figure S31, Supporting Information). Figure 4I shows capacitance retention at a scan rate of 10,000 mV s^−1^. Surprisingly, C‐AAO‐U exhibited the highest retention rate (≈47.1%), outperforming C‐AAO‐G (≈45.9%) and C‐AAO‐S (≈44.9%). Contrary to our initial expectations, the gradient structures did not yield the best performance. EIS data further confirmed this, with C‐AAO‐U showing the shortest τ_0_ (≈0.239 s), compared to C‐AAO‐G (≈0.253 s) and C‐AAO‐S (≈0.260 s) (Figure 4J; Figure S31, Supporting Information). This presents a puzzling result: although transversal nanopores near the interface significantly enhance charging dynamics, a uniform nanopore distribution ultimately delivers superior overall performance.

### 2.4 Finite Element Simulated Charging Process of the 3D C‐AAO Electrodes

**Figure 5:**
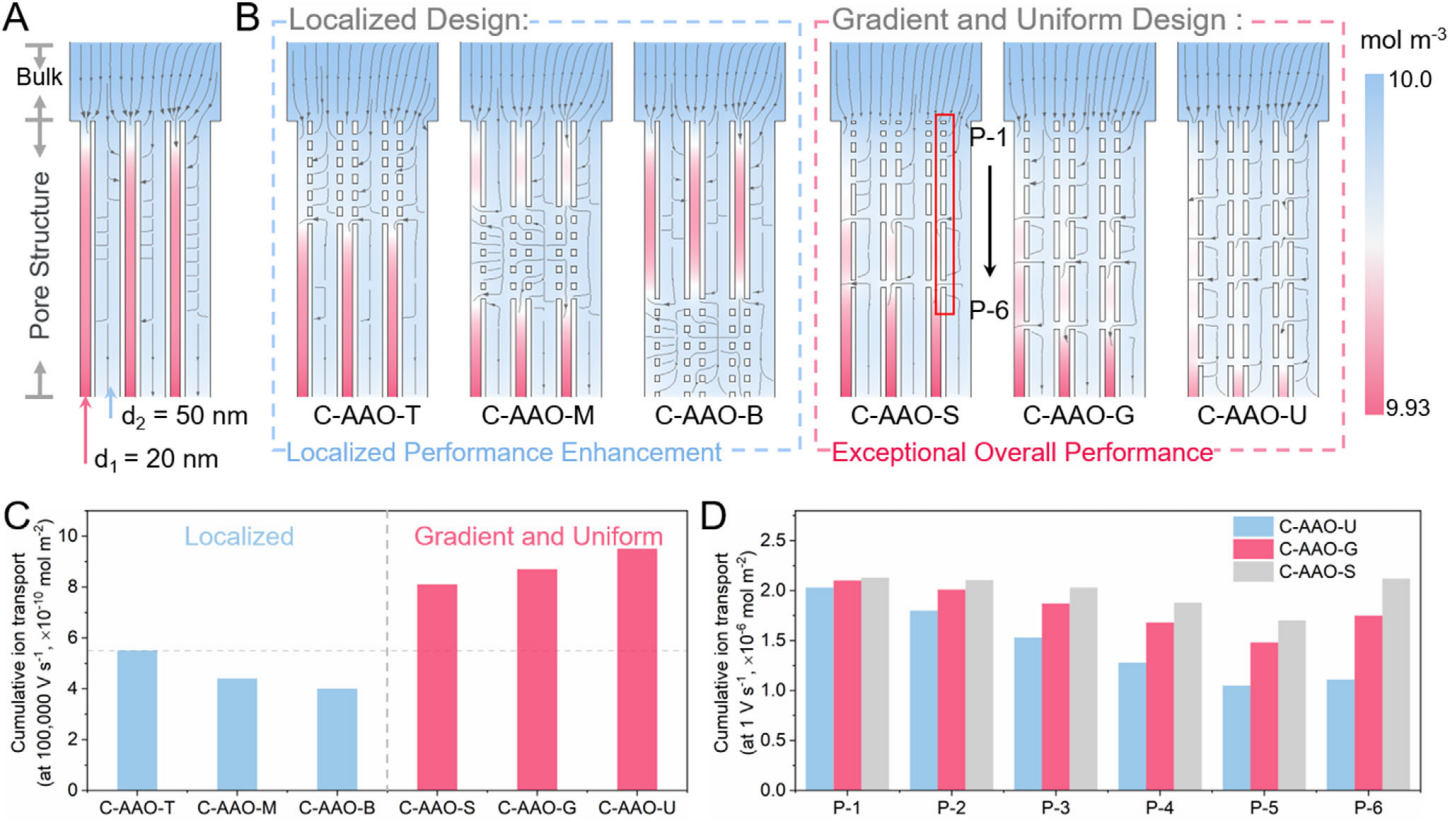
Finite element simulated the charging process of 3D C‐AAO electrodes. A) Ionic concentration distribution maps of the 3D C‐AAO model without transverse pores. B) Ionic concentration distribution maps of the C‐AAO‐T, C‐AAO‐M, C‐AAO‐B, C‐AAO‐U, C‐AAO‐G, and C‐AAO‐S electrodes. a and b share the same color legend. C) Cumulative ion transport rates of transverse pores in the C‐AAO‐T, C‐AAO‐M, C‐AAO‐B, C‐AAO‐U, C‐AAO‐G, and C‐AAO‐S electrodes at 10 0000 V s^−1^. D) Cumulative ion transport rates of the differently located transverse pores in C‐AAO‐U, C‐AAO‐G, and C‐AAO‐S electrodes at 1 V s^−1^. The positions of P‐1 to P‐6 are shown in (B) (Gradient and Uniform Design).

To gain a comprehensive understanding of our findings, we performed finite element simulations to explore the underlying mechanisms. During the charging process, unavoidable variations in nanopore sizes alter the ion concentration distributions within individual nanopores. In narrower pores (d_1_), the limited volume and reduced ion flux lead to rapid ion depletion near the entrance, resulting in pronounced ion depletion.^[^ ^47^ ^]^ In contrast, wider pores (d_2_) enable more uniform ion distribution due to their larger volume and higher ion flux, maintaining concentrations closer to that of the bulk electrolyte (**Figure** **5**A; Figure S32, Supporting Information). The impact of the spatial arrangement of transversal nanopores on ionic concentration distribution was further compared in Figure 5B and Figure S33 (Supporting Information). The results show that the localized design can only alleviate the concentration difference in the corresponding local area, while the gradient and uniform design can effectively reduce the concentration difference in the entire electrode. To quantitatively evaluate the performance improvement of the interconnected porous structure, we analyzed ion flux through the transversal nanopores at a high charging rate (10 0000 V s^−1^, scaled based on size effects from experimental and simulation data to account for ion transport limitations).^[^ ^48^ ^]^ As shown in Figure 5C, C‐AAO‐U demonstrates the highest cumulative ion transport, followed by C‐AAO‐S, C‐AAO‐G, C‐AAO‐T, C‐AAO‐M, and C‐AAO‐B, in descending order. In short, the gradient and uniform design schemes outperform the localized designs, with the uniform scheme demonstrating the greatest performance improvement, consistent with the experimental results in Figure 4.

To investigate the effects of gradient and uniform designs on electrode charging, we further analyzed ion flux through the transversal nanopores at a slower charging rate (1 V s^−1^) (Figure 5D). At this rate, ion diffusion can keep pace with voltage changes, allowing us to treat it as quasi‐steady‐state diffusion. The cumulative ion transport through the transversal nanopores correlates positively with the average ion diffusion distance within the electrode. This relationship suggests that higher cumulative ion transport corresponds to a weaker high‐frequency performance. As shown in Figure 5D, the total ion transport increases with the gradient in the pore structure. Among the tested models, C‐AAO‐U exhibited the lowest cumulative ion transfer in its transversal nanopores compared to C‐AAO‐G and C‐AAO‐S. This finding indicates that during the homogenization of ion concentration within the electrode, a uniform distribution of transversal nanopores minimizes the increase in average ion diffusion distance, enabling the most balanced and efficient ion transport.

## 3 Conclusion

This work introduces a design strategy for 3D carbon nanoporous electrodes with controlled pore densities and spatial distributions. By decoupling ion pathways, we demonstrate that ions prioritize temporal efficiency over spatial proximity, challenging conventional transport models. Uniform nanopore distributions outperform gradient and localized designs, enhancing capacitance retention by 20% at ultrahigh rates. These insights provide a roadmap for developing high‐power electrochemical devices, emphasizing the importance of interconnected porous architectures even in low‐curvature systems.

## Conflict of Interest

The authors declare no conflict of interest.

## Author Contributions

J.L. and L.W. conceived the idea. L.W. supervised the project. J.L. performed the experiments with technical assistance from L.Z., Y.Y., S.Y., L.W., C.Z., Z.Z., and J.H. J.L., and X.Z. performed the finite element method simulations. J.L. and L.W. wrote the manuscript. All authors contributed to the manuscript and approved the final version.

## Supporting information



Supporting Information

## Data Availability

The data that support the findings of this study are available from the corresponding author upon reasonable request.
