# Supplementary material for: Revealing Dynamic Ion Transport in Tailorable Carbon Nano‐Skyscraper Electrodes
Source: Adv Sci (Weinh). 2025 Jun 5;12(32):e03749. doi: 10.1002/advs.202503749 (PMC12407346; doi:10.1002/advs.202503749)
Supplement: Supplementary file 1 — Supporting Information [file ADVS-12-e03749-s001.pdf]

ADVANCED SCIENCE
Open Access

Supporting Information

for *Adv. Sci.*, DOI 10.1002/advs.202503749

Revealing Dynamic Ion Transport in Tailorable Carbon Nano-Skyscraper Electrodes

*Jiye Li, Xiaoyang Zheng, Luting Zhu, Yihang Yao, Sisi Yan, Lang Wang, Jing He, Chen Zhao, Ziqian Zhou and Liaoyong Wen**

Supporting Information

Revealing dynamic ion transport in tailorable carbon nano-skyscraper electrodes

*Jiye Li, Xiaoyang Zheng, Luting Zhu, Yihang Yao, Sisi Yan, Lang Wang, Jing He, Chen Zhao, Ziqian Zhou, Liaoyong Wen**

SUPPLEMENTAL EXPERIMENTAL PROCEDURES

1. Materials and Methods

1.1. Freestanding 3D AAO templates Fabrication

A 0.25 mm thick ultrapure (99.999%) aluminum foil was cut into 2 × 2 cm^2 pieces, and then cleaned and degreased by sonication in acetone, water, isopropanol, and ethanol. After cleaning, the foil was placed in an electrical polishing solution ($HClO_4/C_2H_5OH$, 1:4 in volume ratio) and subjected to a 20 V positive voltage for 3 minutes. The polished aluminum foil was then immersed in an anodizing solution (0.3 M H_2SO_4) at 0 °C, with a 25 V voltage applied between the aluminum foil and the negative graphite electrode. After anodizing for 24 hours, the anodic layer was removed by chemical etching in a mixture of phosphoric acid (7 wt%) and chromic oxide (1.8 wt%). The etched aluminum foil was then pulse anodized according to a predefined pulse program (The constant voltage stage was set at 25 V, while the pulse treatment was applied at 34 V. Each pulse lasted for 1 s, with a limiting current of 20 mA cm^{-2}. The detailed pulse program used for the 3D AAO templates in this study is summarized in Table S1.). This process resulted in 3D AAO templates supported by the aluminum plate. Next, electrochemical stripping was performed in a stripping solution ($HClO_4$: C_2H_5OH = 1:1 in volume ratio) at a voltage of 30 V for three minutes to obtain freestanding 3D AAO templates with through-holes. Finally, the freestanding 3D AAO templates were submerged into a 10 wt% aqueous H_3PO_4 solution at 1°C to create the transversal nanochannels.

1.2. Preparation of the 3D C-AAO films

The 3D AAO templates were first cut into circles with diameters of 1 cm using a laser (UNIVERSAL VLS3.50). These templates were then treated at 750 °C for 24 hours under a load of three flat silicon wafers to eliminate the internal stress and reduce the deformation during the subsequent CVD process. The 3D C-AAOs were prepared by depositing a uniform carbon coating on 3D AAO templates through a CVD process. In this process, the furnace was heated to 620 °C at a rate of 10 °C per minute while maintaining a continuous flow of argon gas at 200 SCCM (standard cubic centimeter per minute). Once the furnace reached 620 °C, a mixture of hydrogen (10 SCCM) and acetylene (20 SCCM) was introduced. The 3D AAO templates were held in the furnace for 1 hour to allow the carbon coating to growth. After the growth process was completed, the furnace was cooled down to room temperature while continuing the flow of argon gas. Following cooling, the 3D C-AAO films were taken out from the quartz tube for characterization and directly used as electrodes without any further treatment.

1.3. Assembly of button-type supercapacitors

A commercially available 2032-type coin cell kit was used to assemble the supercapacitor. Each supercapacitor consisted of two 3D C-AAO electrodes, an electrolyte, and a separator (Whatman GF/D). The electrolytes used in this study were:

- butyl-3-methylimidazolium tetrafluoroborate (Emim-BF_4)
- Emim-BF_4 in acetonitrile solution (1 M)
- Sodium sulfate aqueous solution (0.5 M)

To enhance the conductivity between the electrodes and the current collector and to minimize contact resistance, gold-plated aluminum foil (50 nm thickness of Au) was used as the current collector.

2. Characterizations

2.1. SEM and TEM

The scanning electron microscopy (SEM) images were obtained using an Analytical Field Emission Scanning Electron Microscope (Zeiss Gemini 450). Transmission electron microscopy (TEM) was conducted using a high-resolution transmission electron microscope (Talos F200X G2).

2.2. 3D C-AAO geometry analysis

Random regions of the 3D C-AAOs were imaged at a specific magnification (20,000×) using SEM and analyzed with ImageJ (open-source software, https://imagej.nih.gov/ij/).

✧ The diameter D of the circular pores can be calculated using the following equation:

$$D = 2\sqrt{\frac{A_{s-pore}}{\pi}} \quad (1)$$

where $A_{s\text{-}pore}$, is the area of a single pore.

✧ The pore density P of the electrode can be determined using the equation:

$$P = \frac{N_{count}}{A_{total}} \quad (2)$$

where N_{count} is the total number of pores and A_{total} is the total area. These values can be directly obtained from the SEM images using ImageJ.

2.3. Electrochemical measurements

Electrochemical performances were evaluated using a BioLogic VSP-128 electrochemical station, employing techniques such as cyclic voltammetry (CV), galvanostatic charge-discharge (GCD), and electrochemical impedance spectroscopy (EIS). EIS measurements were conducted in the frequency range of 100 mHz to 100 kHz with an amplitude of 10 mV. Considering temperature stability, all electrochemical measurements were performed at 25 °C.

✧ The capacitance C of an electrode is calculated from CV data using the following equation:

$$\mathrm{C} = \frac{\int I\,\mathrm{d}V}{2v\Delta V} \quad (3)$$

✧ The areal capacitance C_A is determined using the equation:

$$C_A = \frac{C}{A_{\mathrm{ele}}} \quad (4)$$

where I is the current (mA), ΔV is the scan potential range (V), v is the scan rate (V s^{-1}), and A_{ele} is the surface area of the electrode (cm^2).

✧ The relaxation time constant τ_0 is calculated using the equation:

$$\tau_0 = 1/f_0 \tag{5}$$

Where f_0 is the frequency at which the phase degree is 45°.

2.4. Ionic conductance and Ion diffusion coefficient measurements

I-V curves were measured using a source meter (Agilent B2912A). Two Ag/AgCl electrodes were employed to apply the potential. Silicone gaskets clamp the porous membrane during testing and ensure a uniform testing area. The diameter of the central pore of the silicone gasket is 0.2 cm.

✧ The Specific Conductance σ is calculated by the equation:

$$\sigma = \frac{\mathrm{G\,L}}{\mathrm{A}} = \frac{4\,\mathrm{G\,L}}{\mathrm{N}\,\pi d^2} \tag{6}$$

where G is the total conductance of the membrane(S) (obtained from the slope of the I-V curves), A *is* the cross-sectional area of the pore (cm^2), d is the diameter of each pore (cm), N is the total number of pores in the membrane, and L is the distance (cm) between the two Ag/AgCl electrodes.

✧ The Ion diffusion coefficient D is calculated using the multi-ion Nernst-Einstein equation:

$$\sigma = \frac{F^2}{RT}\sum_i z_i^2 C_i D_i \tag{7}$$

Where D is the diffusion coefficient of the ion, σ is the ionic conductivity, R is the gas constant, T is the temperature, F is the Faraday's constant, z is the charge of the ion, c is the concentration of the ion, i is the number of ion species.

2.5. COMSOL simulation

The dynamic ion behaviors in different 3D C-AAO electrodes were investigated using a finite element simulation platform, COMSOL Multiphysics (Version 6.2, COMSOL, Sweden). We used a 2D model with the electroanalysis module to calculate the diffusion of an EMIM-BF_4 electrolyte using Fick's law, and the local mass balances at the electrode surfaces of 3D C-AAO.

The ion A adsorbs to the 3D C-AAO electrode surfaces according to

$$A \leftrightarrow A_{ads}$$

In the electrode surfaces, Langmuir isotherms are used to describing the kinetics, with the adsorption rate defined as

$$r_{ads} = k_a c_A\left(1-\theta_{A_{ads}}\right) - k_d\theta_{A_{ads}}$$

where $k_a = 10^{-5}$ m s^{-1} is the adsorption rate constant, c_A is the concentration of species A in the electrolyte, $\theta_{A_{ads}}$ is the surface coverage of species A on the electrode, and $k_d = 10$ mol m^{-2} s^{-1} is the desorption rate constant. The initial surface coverage of species A is set to $\theta_{A_{ads}} = 0$.

The charge transfer reaction in the electrode surfaces is defined as

$$i_{loc} = -k_0\Gamma F\left(\theta_{A_{ads}} e^{-\frac{0.5\eta F}{RT}}\right)$$

where $k_0 = 100$ is charge transfer rate constant, $\Gamma = 0.1$ mol·m^{-2} is density of surface sites at the electrode, $F = 96485$ C·mol^{-1} is Faraday's constant, $R = 8.3145$ J·mol^{-1}·K^{-1} is molar gas constant, and $T = 293$ K is temperature.

The overpotential η is defined as

$$\eta = E - E_0$$

where E is the electrode potential and E_0 the formal potential.

The time-dependent governing equation of the reaction rate of species A in the electrolyte is defined as:

$$\frac{\partial C_A}{\partial t} + \nabla \cdot \boldsymbol{J}_A + \nabla c_A = R_A$$

$$\boldsymbol{J}_A = -D_A \nabla c_A$$

where $D_A = 1 \times 10^{-9}$ m^2 s^{-1} is the diffusion coefficient of species A.

In addition, the external electric potential was set as to 100,000 V s^{-1} or 1 V s^{-1}, and the initial ion concentration was set as to 0.01 M.

SUPPLEMENTAL FIGURES

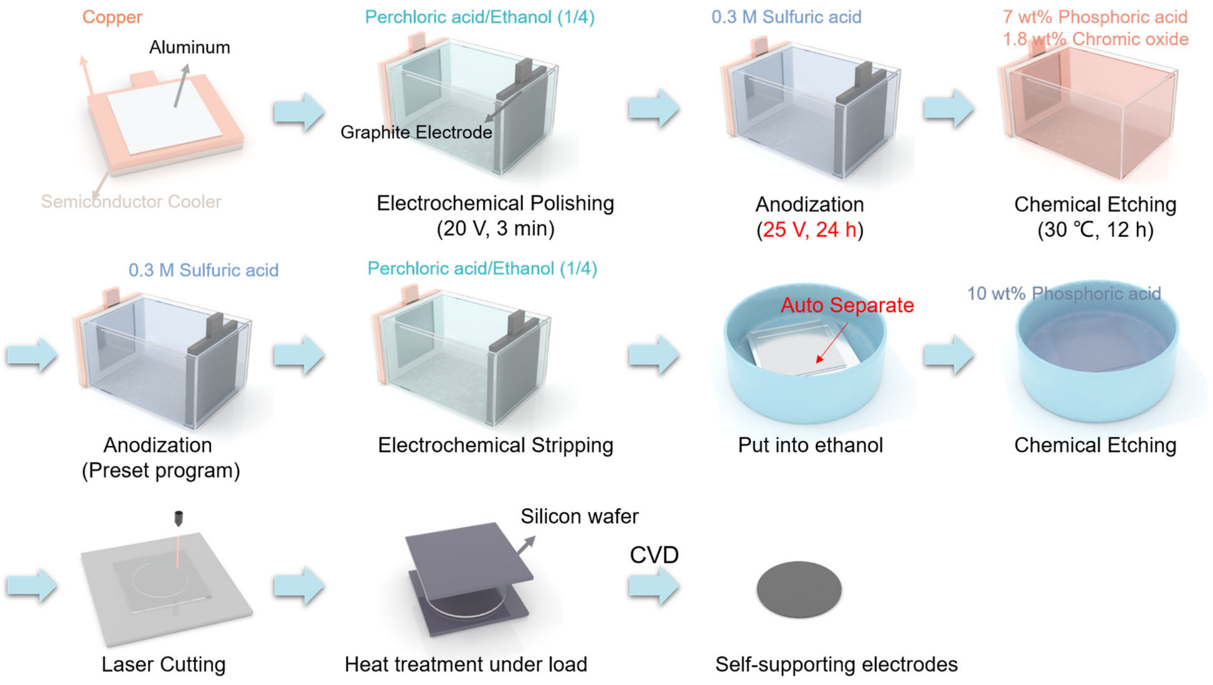

Figure S1. Schematic diagram illustrating the preparation process for 3D C-AAO. Initially, 3D AAO templates are fabricated via a two-step anodization process using a polished aluminum sheet. Electrochemical stripping, laser cutting, and heat treatment are then applied to the 3D AAO templates. Finally, a carbon film is deposited on the AAO template using chemical vapor deposition (CVD).

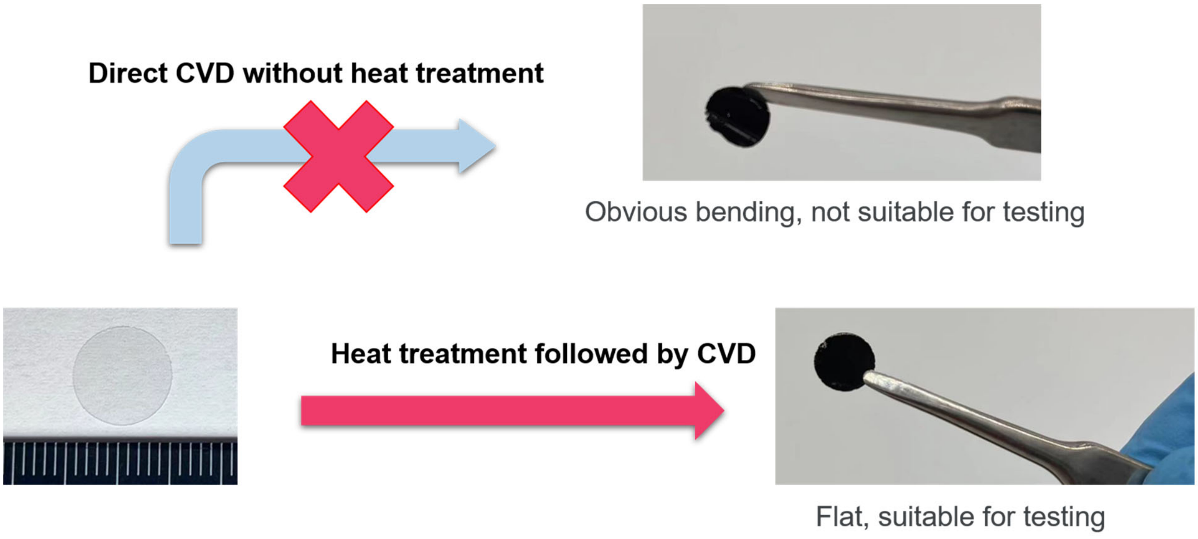

Figure S2. Importance of heat treatment under load.

Note of Figure S2: Relieving the internal stress of 3D AAO during the preheating process prevents bending during the subsequent CVD process.

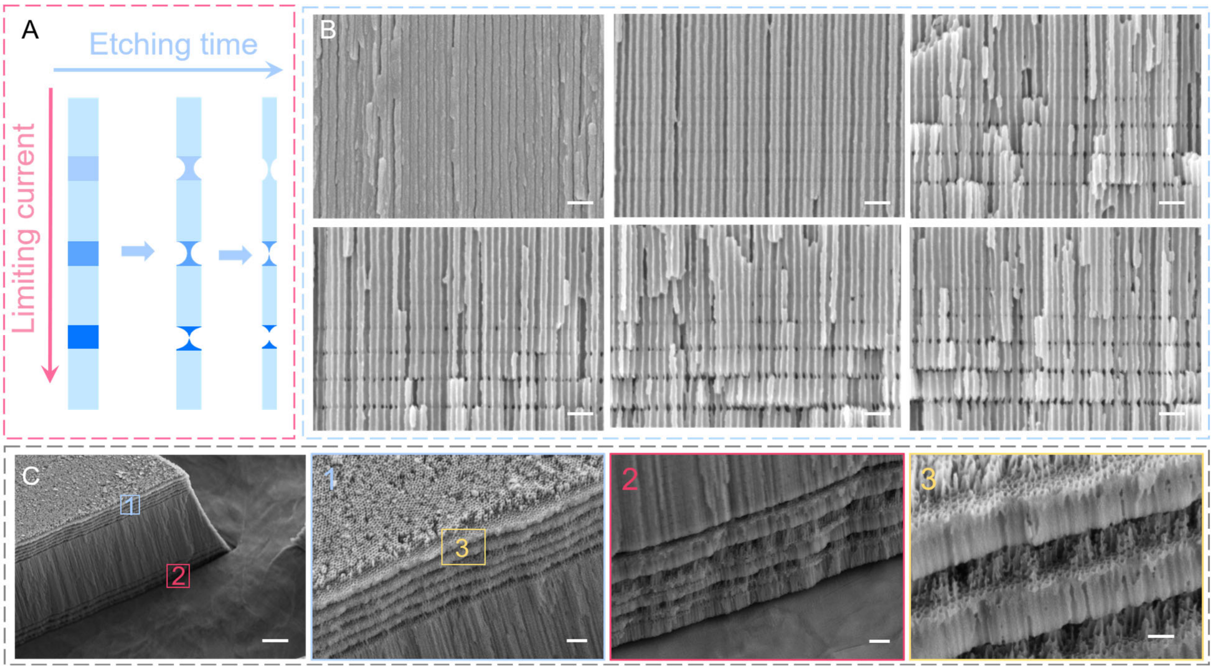

Figure S3. Controlled construction of transversal pores.

(A) Schematic diagram showing the effects of limiting pulse current and etching time on transversal pores in 3D AAO. The transversal pores are formed based on the etch rate difference between AAO produced by constant voltage anodization and pulse anodization. Increasing the limiting current enhances the etch rate difference between the two anodization methods.

(B) Cross-sectional SEM of 3D AAO with different etching times (increased from 0 h to 7 h). The limiting current gradually increases during the pulse process (from 0.16 mA cm^{-2} to 0.44 mA cm^{-2}).

(C) When the current is not limited, the pulse process becomes uncontrolled, and the thickness of the resulting AAO cannot be accurately controlled.

Scale bars: (A) 200 nm; (B) 2 μm, 400 nm, 400 nm, 200 nm.

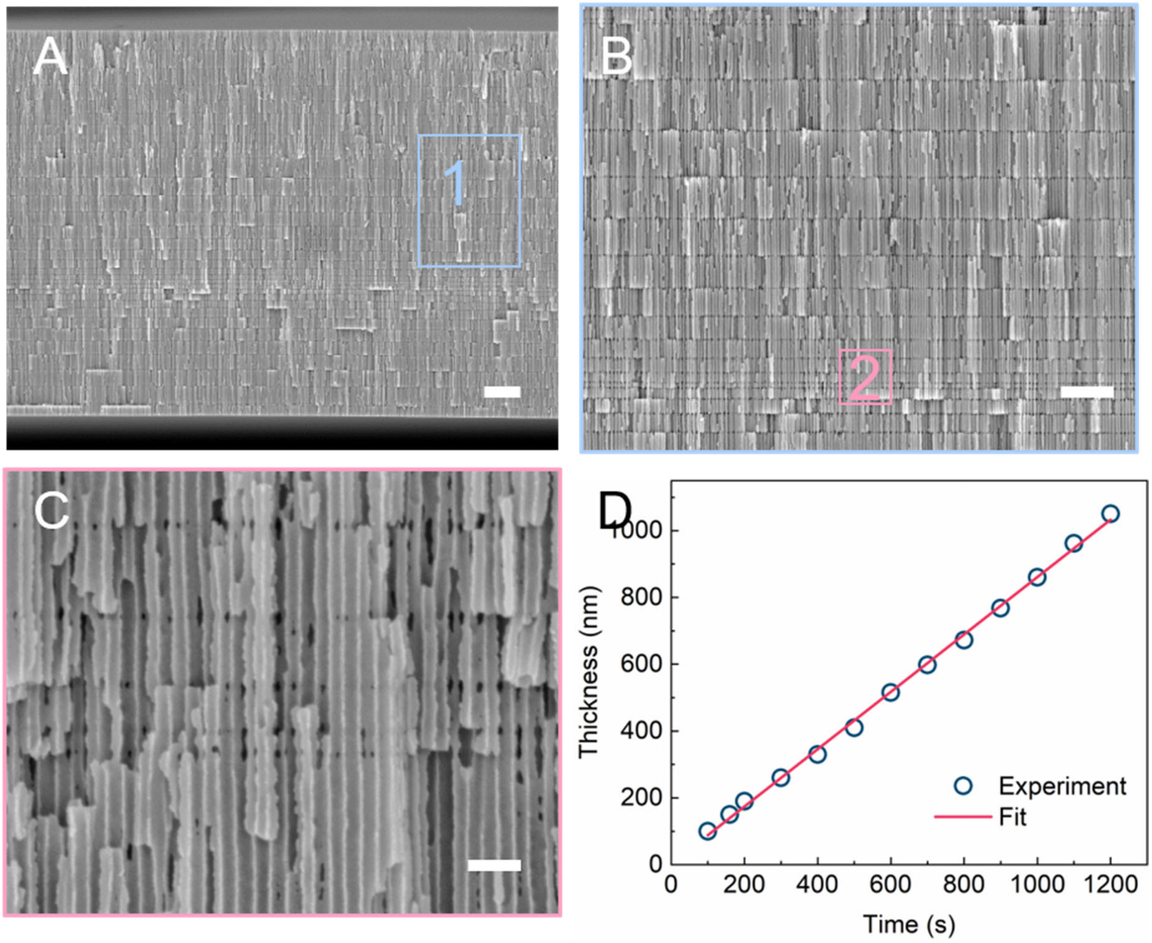

Figure S4. Relationship between transversal pore spacing and anodizing time.

(A) Cross-sectional SEM image of 3D AAO with different pulse time intervals.

(B) Magnified cross-sectional SEM of area 1.

(C) Magnified cross-sectional SEM of area 2.

(D) Relationship between transversal pores spacing and anodizing time.

Scale bars: (A) 2 μm; (B) 1 μm; (C) 200 nm.

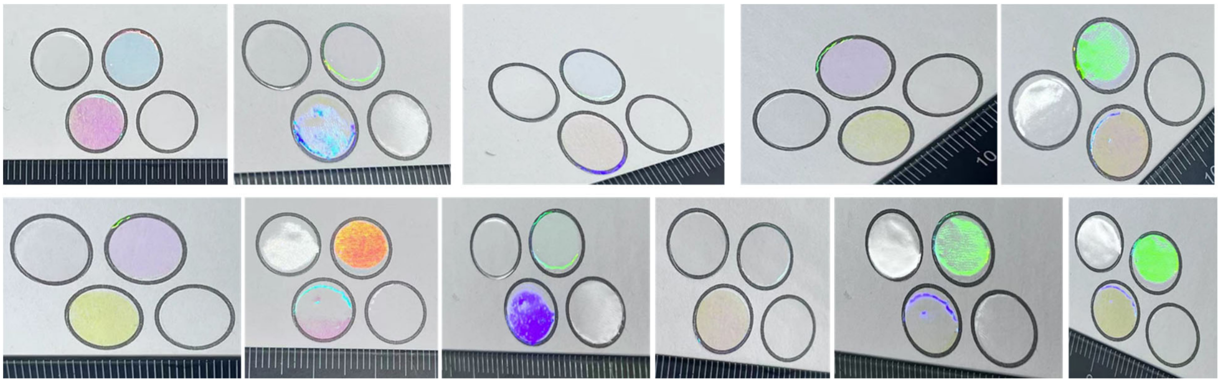

Figure S5. Optical photos of 3D AAO precursors for C-AAO-0, C-AAO-515, C-AAO-225, and C-AAO-150 from different angles.

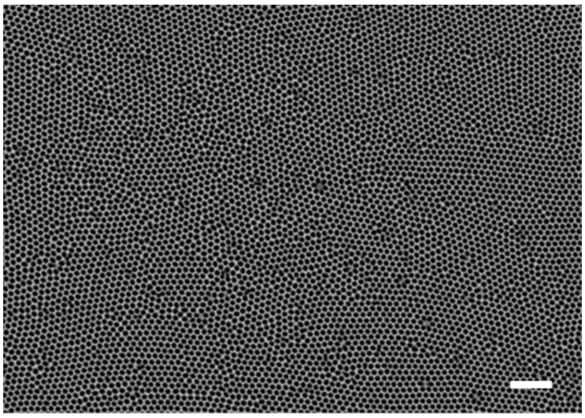

Figure S6. SEM of the bottom surface of the C-AAO-515 template. Scale bars: 400 nm.

Note of Figure S6: The electrochemical stripping of anodized aluminum oxide is a selective etching process conducted under an applied electric field, which does not compromise the structural order or pore morphology of the AAO membrane.

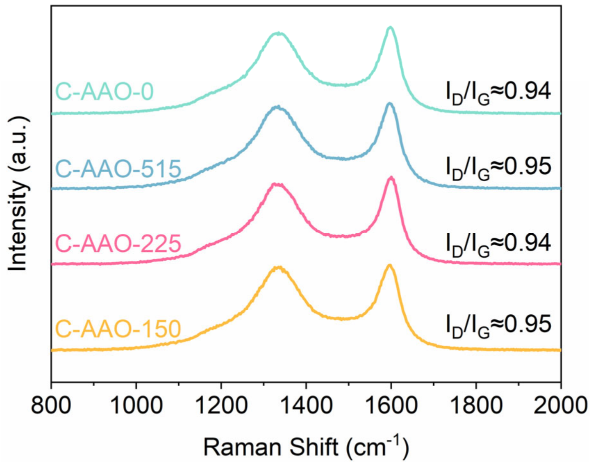

Figure S7. Raman spectra of C-AAO-0, C-AAO-515, C-AAO-225 and C-AAO-150.

Note of Figure S7: The I_D/I_G ratios of the C-AAO-0, C-AAO-515, C-AAO-225, and C-AAO-150 are nearly identical, indicating comparable structural order. These results suggest that variations in the AAO structure have minimal influence on the ordering of the deposited carbon layer.

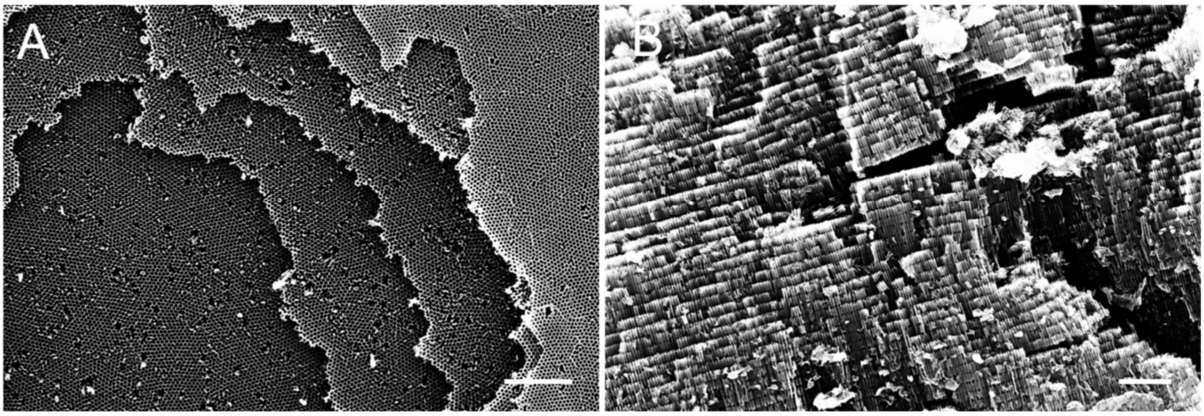

Figure S8. SEM images of C-AAO-225 after removing 3D AAO template.

(A) Top view.

(B) Side view.

Scale bars: (A) 1 μm; (B) 1 μm.

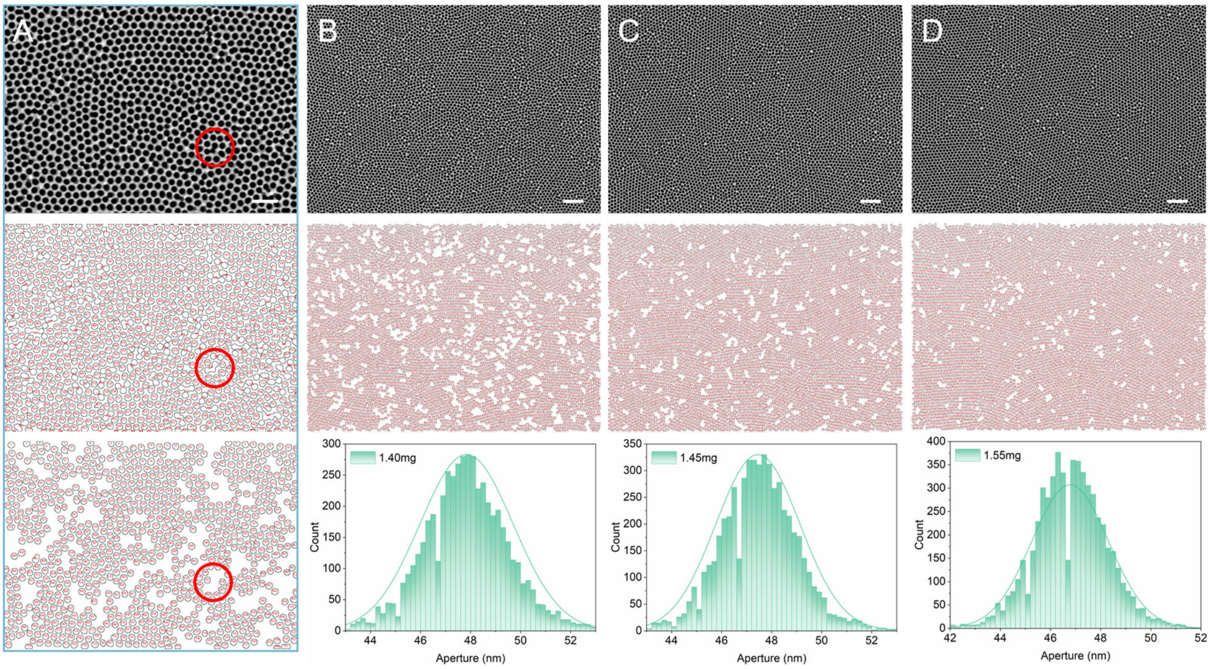

Figure S9. SEM images and pore size distribution plots of C-AAO-0 with different weights.

(A) Pore size of C-AAO-0 was analyzed and converted using ImageJ software. As indicated by the red circle in the SEM image, certain damaged or interconnected pores resulting from etching were excluded from the analysis.

(B) Sample weight: 1.40 mg.

(C) Sample weight: 1.45 mg.

(D) Sample weight: 1.55 mg.

The pore size of C-AAO-0 conforms to a normal distribution.

Scale bars: (A) 200 nm; (B-D) 400 nm.

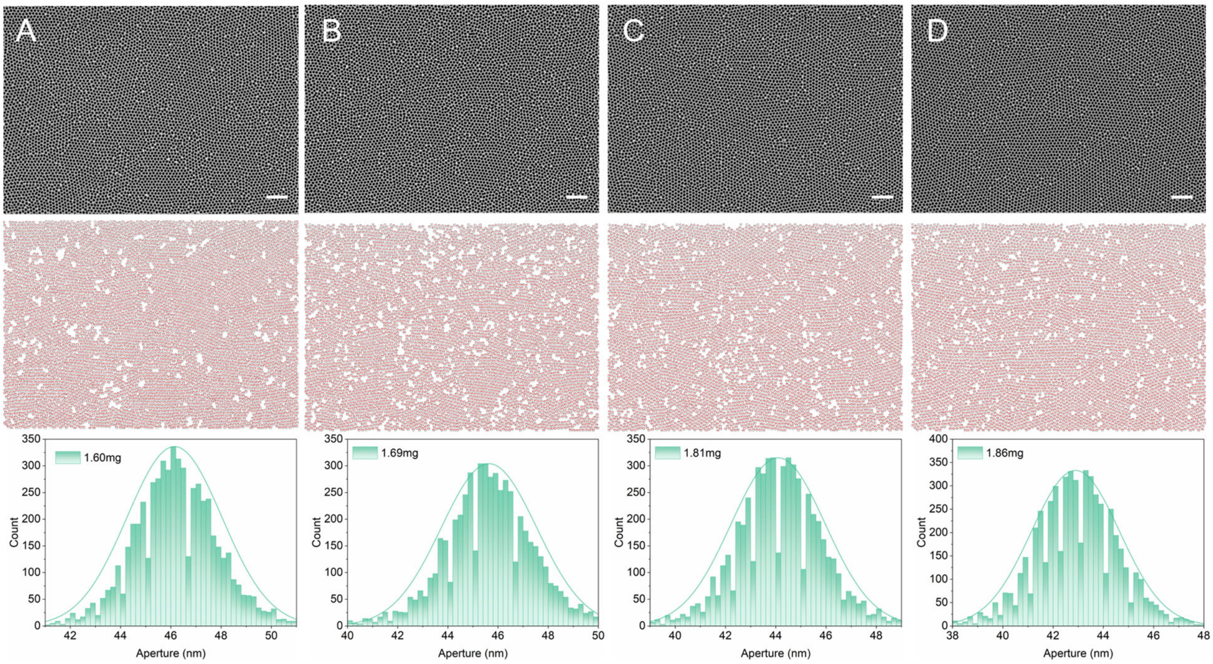

Figure S10. SEM images and pore sizes distribution plots of C-AAO-0 with different weights.

(A) Sample weight: 1.60 mg.

(B) Sample weight: 1.69 mg.

(C) Sample weight: 1.81 mg.

(D) Sample weight: 1.86 mg.

Scale bars: (A – D) 400 nm.

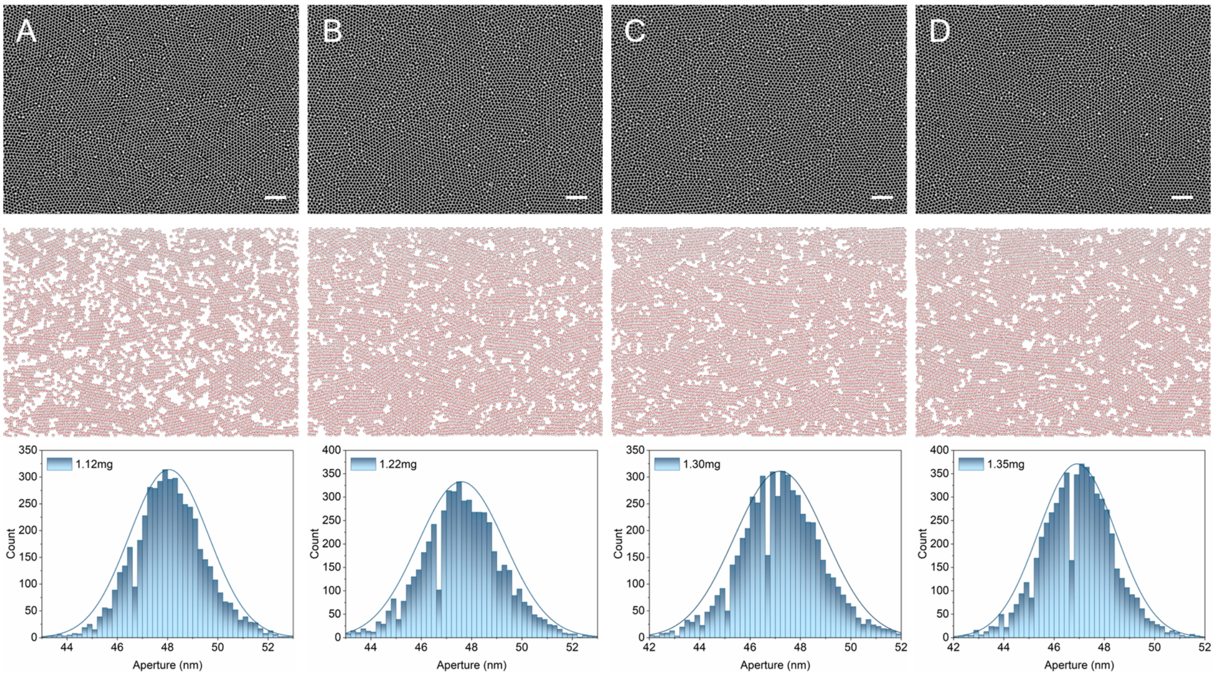

Figure S11. SEM images and pore sizes distribution plots of C-AAO-515 with different weights.

(A) Sample weight: 1.12 mg.

(B) Sample weight: 1.22 mg.

(C) Sample weight: 1.30 mg.

(D) Sample weight: 1.35 mg.

Scale bars: (A – D) 400 nm.

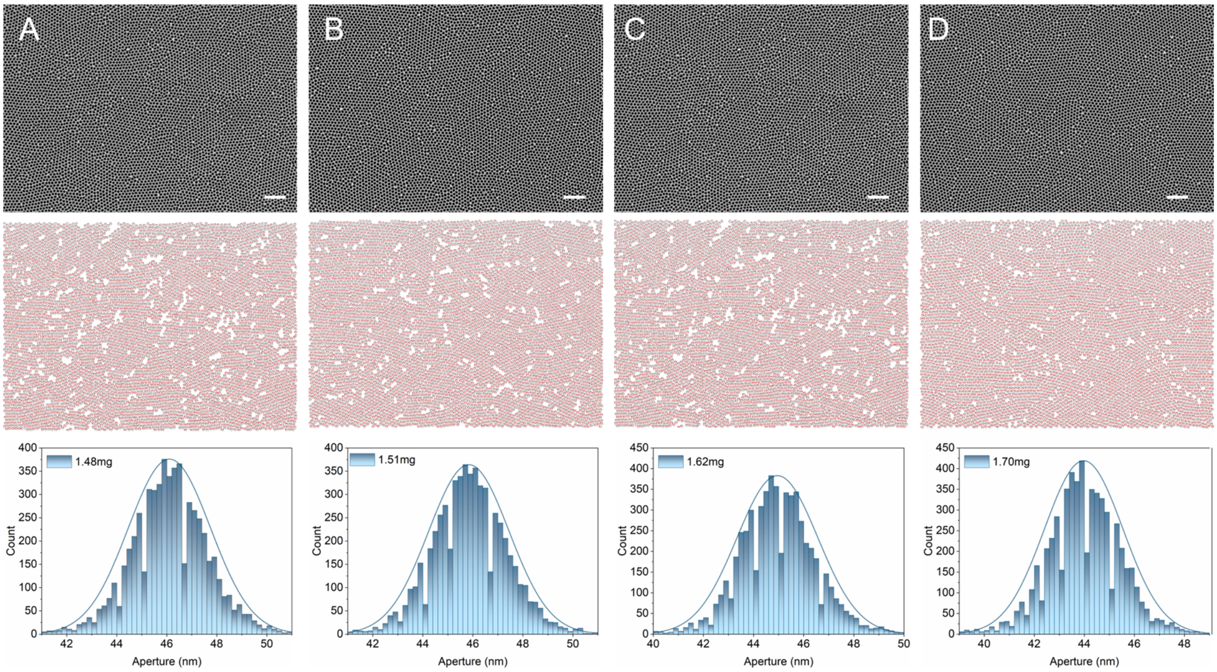

Figure S12. SEM images and pore sizes distribution plots of C-AAO-515 with different weights.

(A) Sample weight: 1.45 mg.

(B) Sample weight: 1.51 mg.

(C) Sample weight: 1.62 mg.

(D) Sample weight: 1.70 mg.

Scale bars: (A – D) 400 nm.

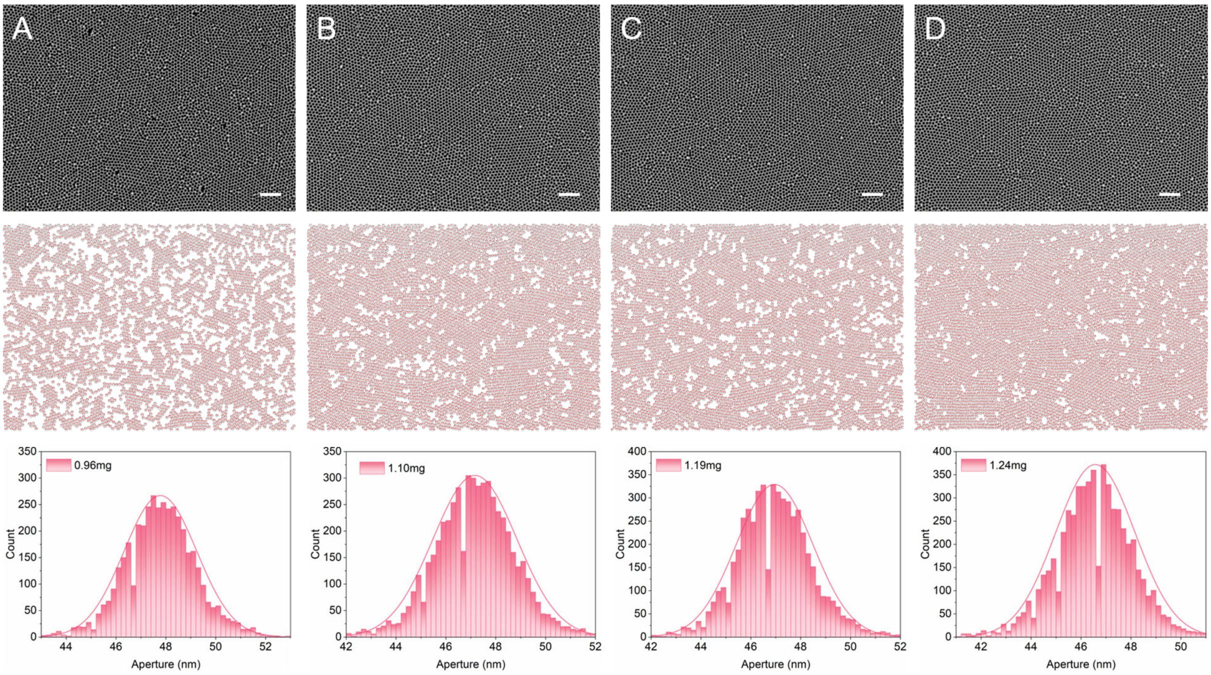

Figure S13. SEM images and pore sizes distribution plots of C-AAO-225 with different weights.

(A) Sample weight: 0.96 mg.

(B) Sample weight: 1.10 mg.

(C) Sample weight: 1.19 mg.

(D) Sample weight: 1.24 mg.

Scale bars: (A – D) 400 nm.

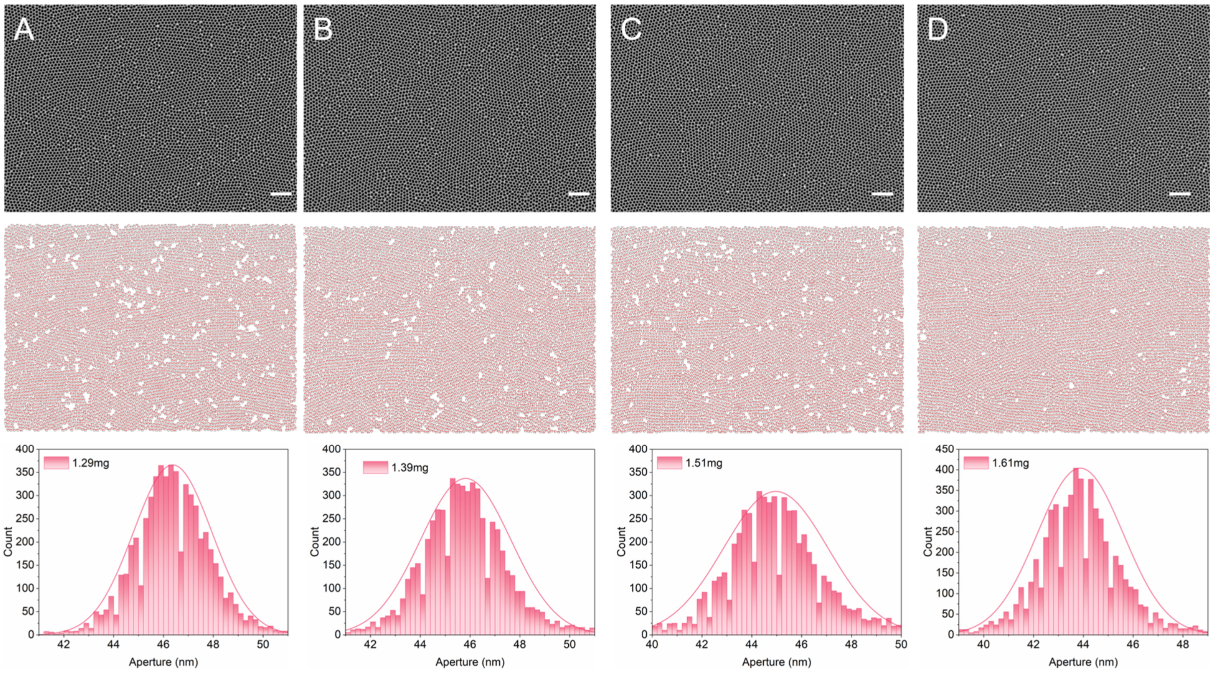

Figure S14. SEM images and pore sizes distribution plots of C-AAO-225 with different weights.

(A) Sample weight: 1.29 mg.

(B) Sample weight: 1.39 mg.

(C) Sample weight: 1.51 mg.

(D) Sample weight: 1.61 mg.

Scale bars: (A – D) 400 nm.

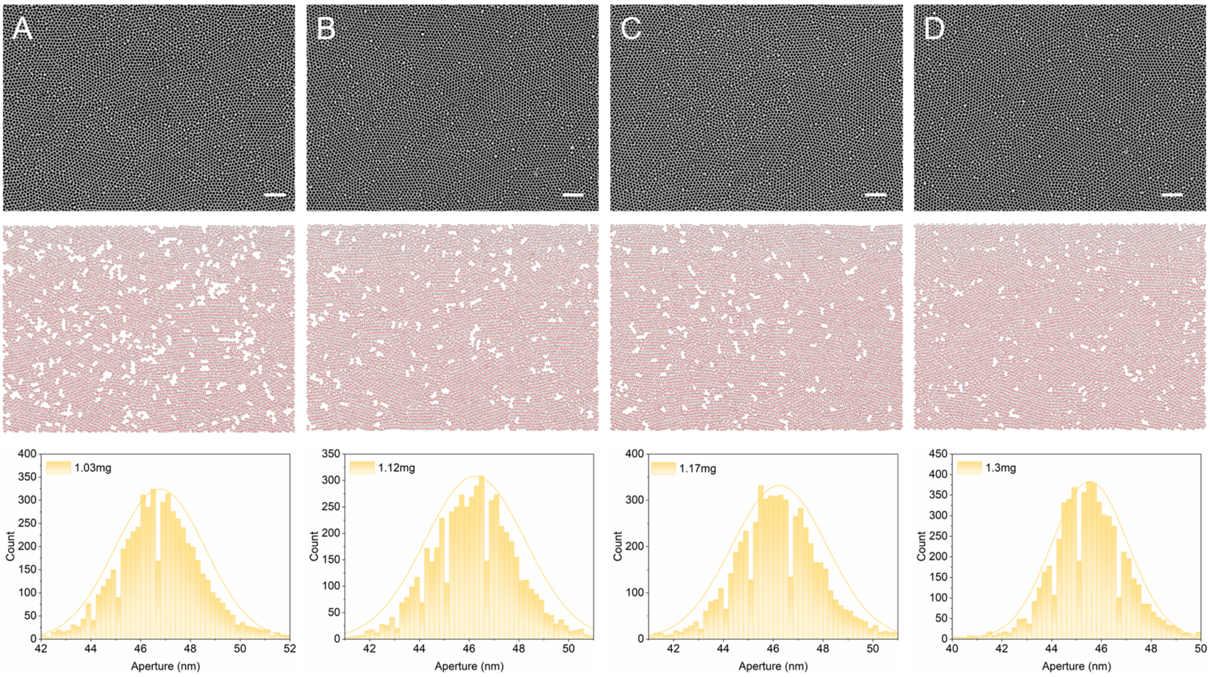

Figure S15. SEM images and pore sizes distribution plots of C-AAO-150 with different weights.

(A) Sample weight: 1.03 mg.

(B) Sample weight: 1.12 mg.

(C) Sample weight: 1.17 mg.

(D) Sample weight: 1.30 mg.

Scale bars: (A – D) 400 nm.

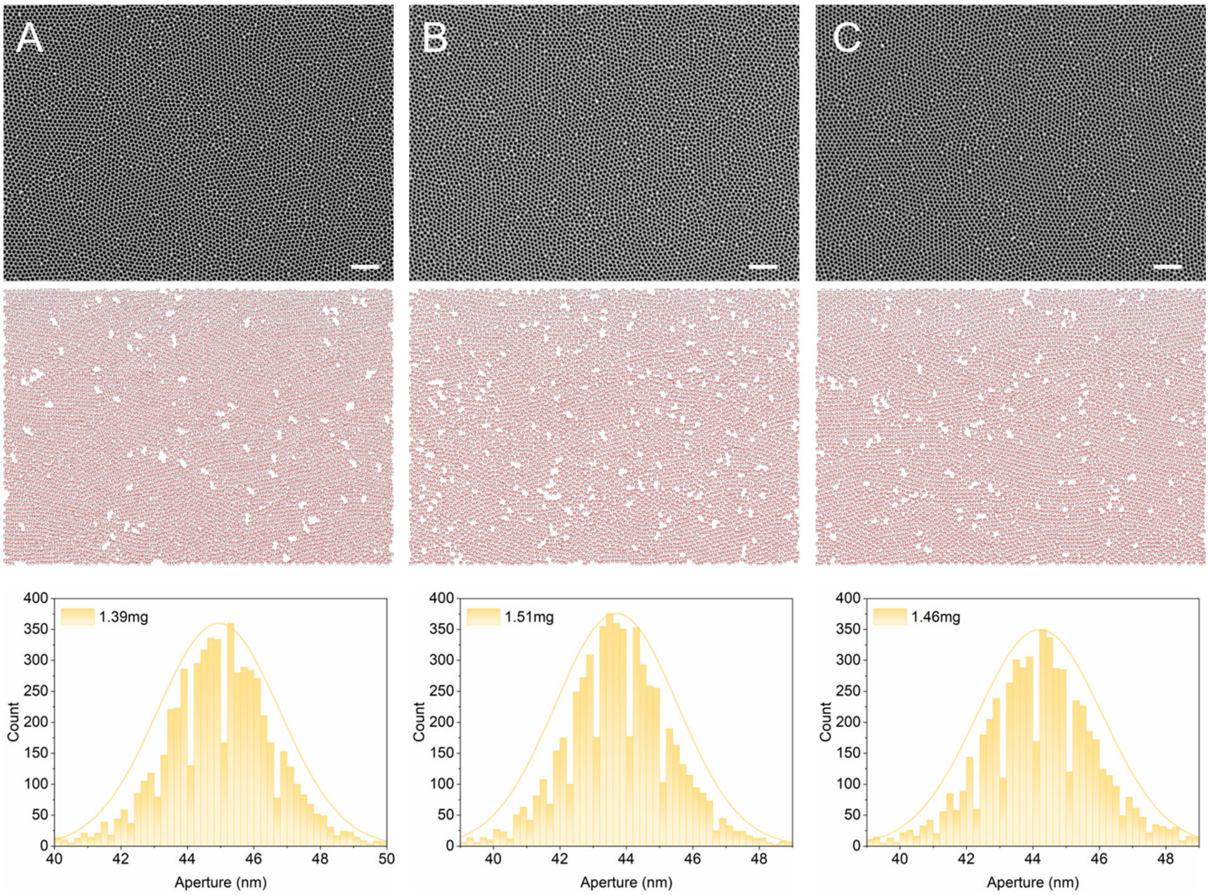

Figure S16. SEM images and pore sizes distribution plots of C-AAO-150 with different weights.

(A) Sample weight: 1.39 mg.

(B) Sample weight: 1.46 mg.

(C) Sample weight: 1.51 mg.

Scale bars: (A – C) 400 nm.

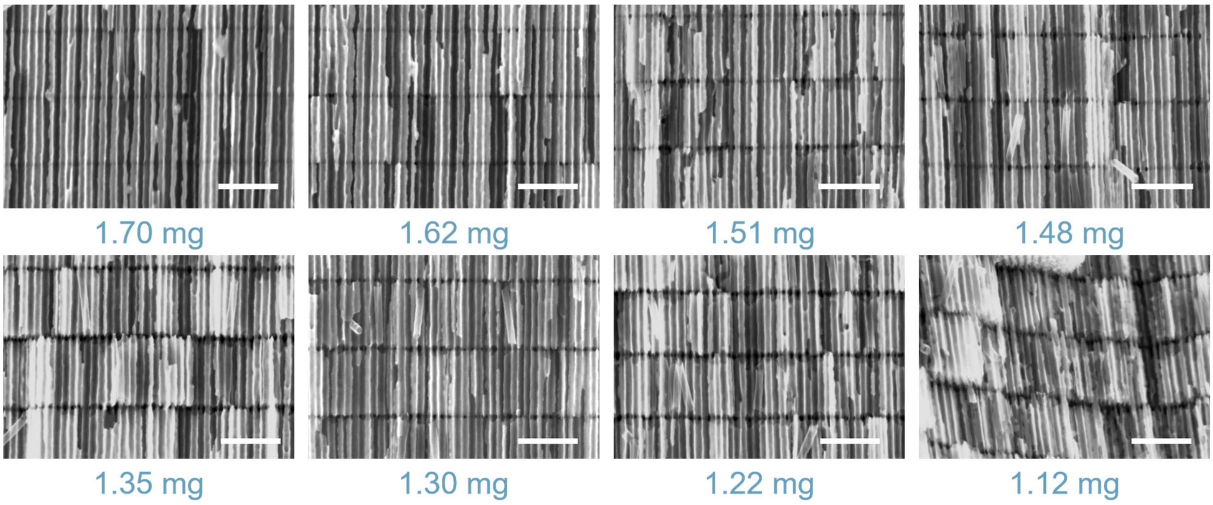

Figure S17. Cross-sectional SEM images of C-AAO-515 with different weights.

The electrode masses are 1.70 mg, 1.62 mg, 1.51 mg, 1.48 mg, 1.35 mg, 1.30 mg, 1.22 mg, and 1.12 mg respectively.

Scale bars: 200 nm.

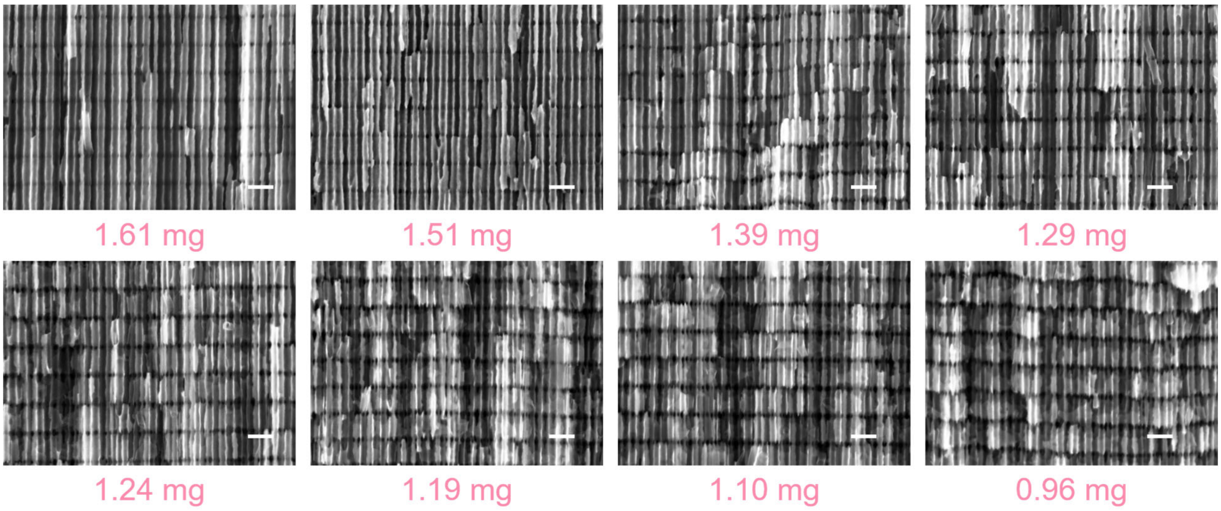

Figure S18. Cross-sectional SEM images of C-AAO-225 with different weights.

The electrode masses are 1.61 mg, 1.51 mg, 1.39 mg, 1.29 mg, 1.24 mg, 1.19 mg, 1.10 mg, and 0.96 mg respectively.

Scale bars: 200 nm.

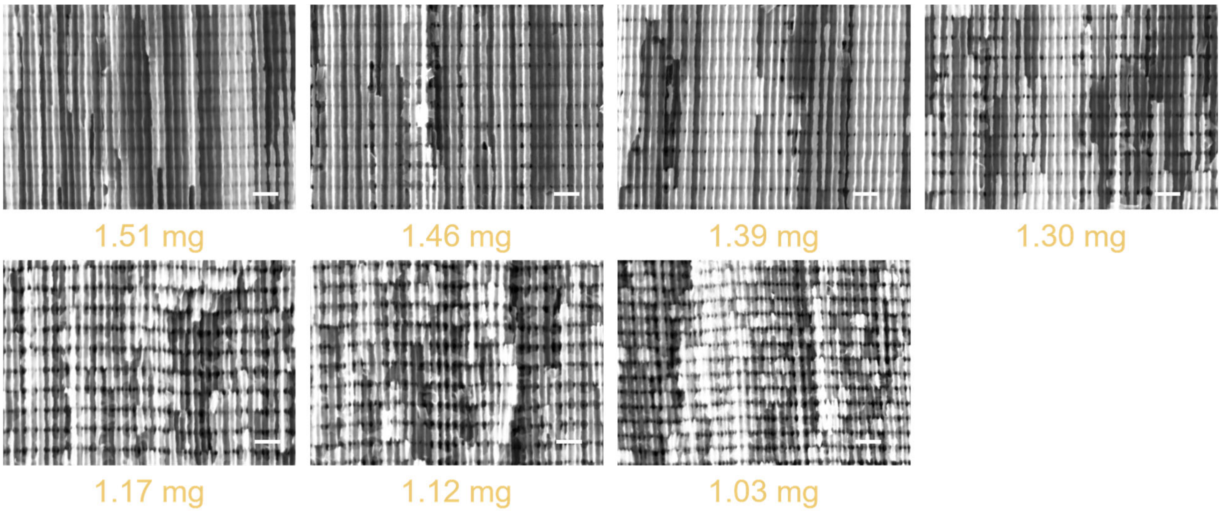

Figure S19. Cross-sectional SEM images of C-AAO-150 with different weights.

The electrode masses are 1.51 mg, 1.46 mg, 1.39 mg, 1.30 mg, 1.17 mg, 1.12 mg, and 1.03 mg respectively.

Scale bars: 200 nm.

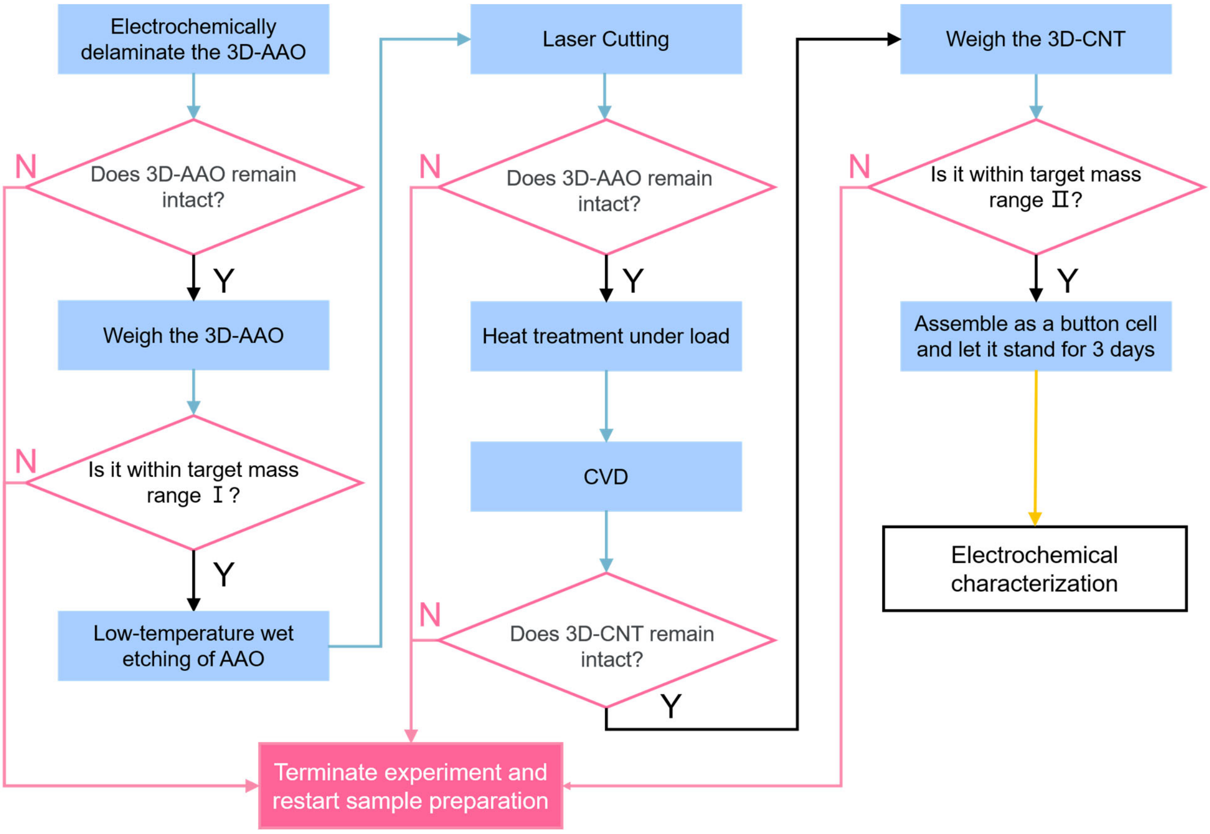

Figure S20. Preparation flow chart of 3D C-AAO electrodes.

Note of Figure S20: We commenced by characterizing the structure of the 3D AAO precursor and 3D C-AAO, followed by measurements of samples with specific structural features. These structural features were then correlated with the sample's mass, transforming abstract structural analysis into a more intuitive process of mass measurement. Multiple quality assessments were conducted to ensure the stability and reliability of our electrode fabrication process. Our measurements indicate that the mass of the newly fabricate(D) unetched 3D AAO with a thickness of 19 μm is 11.70 mg. For Weight Range I, the acceptable mass is 11.70 ± 0.05 mg. Ensuring consistent thickness of the AAO template and controlling the same mass is equal to the pore volume to be the same. The mass error for Ranges II was kept within ± 0.02 mg.

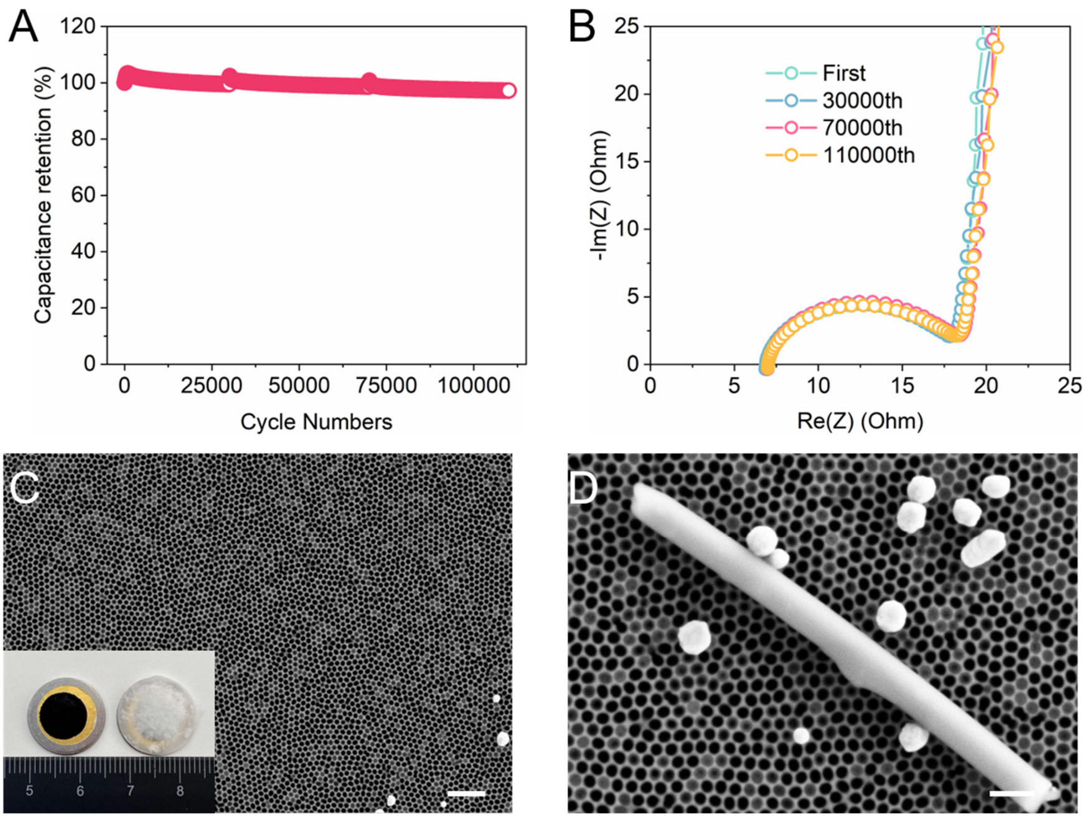

Figure S21. Electrochemical and Mechanical stability of C-AAO.

(A) Cycle performance of C-AAO-515 in pure ionic liquid, which exceeding 110,000 charge/discharge cycles at a current density of 10 A cm^{-2}.

(B) Nyquist plots of C-AAO-515 during cycling.

(C) After cycle testing, SEM image of the disassembled electrode. Inset: Optical image of the disassembled electrode.

(D) Enlarged SEM of the electrode after disassembly. The white rods in the picture are glass fibers.

Scale bars: (C) 400 nm; (D) 200 nm.

Note of Figure S21: After 110,000 charge/discharge cycles, the specific capacitance and impedance of the representative C-AAO-515 electrode remained virtually unchanged. Additionally, post-cycling structural analysis confirmed that the electrode's microstructure was well preserved, indicating that the C-AAO electrode possesses excellent electrochemical and mechanical stability.

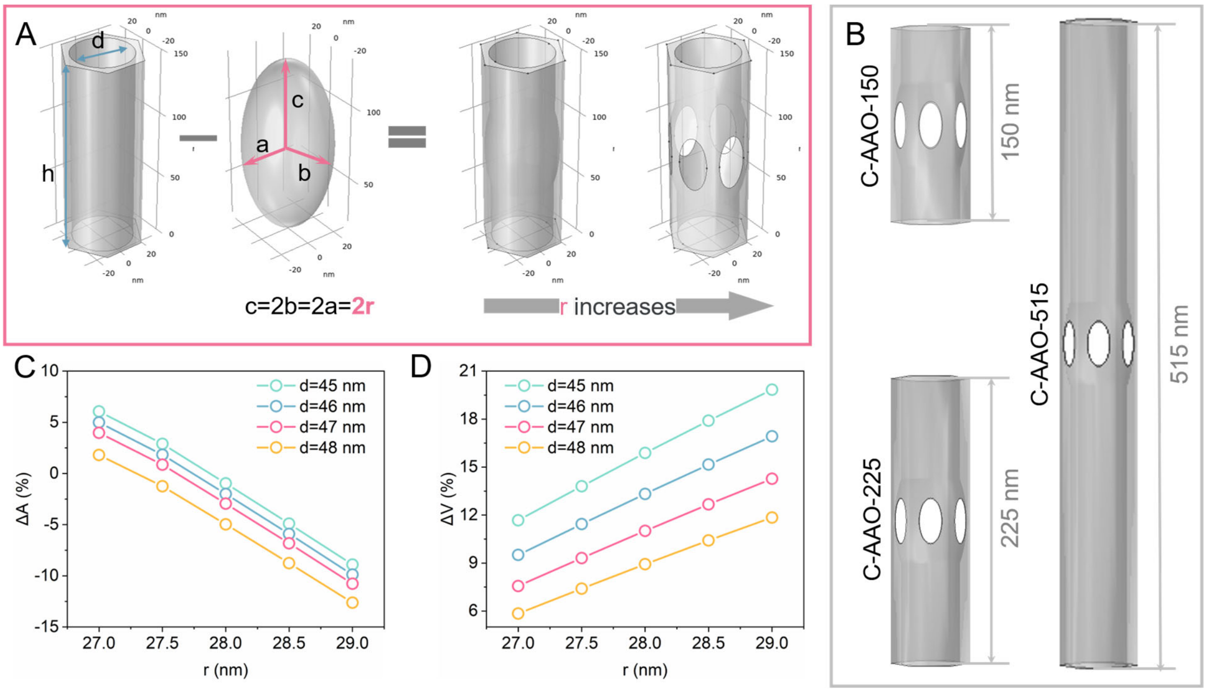

Figure S22. Geometric analysis of the impact of transversal pore construction on the internal surface area and volume of 3D C-AAO.

(A) A representative unit of 3D C-AAO was simulated by Boolean subtraction of an ellipsoid from a hexagonal hollow column. H denotes the pore length, D is the diameter of the inscribed circle, and a, b and c are the three axes of the ellipsoid, where $c = 2b = 2a = 2r$.

(B) Representative unit cells for C-AAO-150, C-AAO-225, and C-AAO-515 are shown.

(C) Effect of variations in inscribed circle and ellipsoid dimensions on the internal surface area of the material.

(D) Effect of variations in inscribed circle and ellipsoid dimensions on the internal volume of the material.

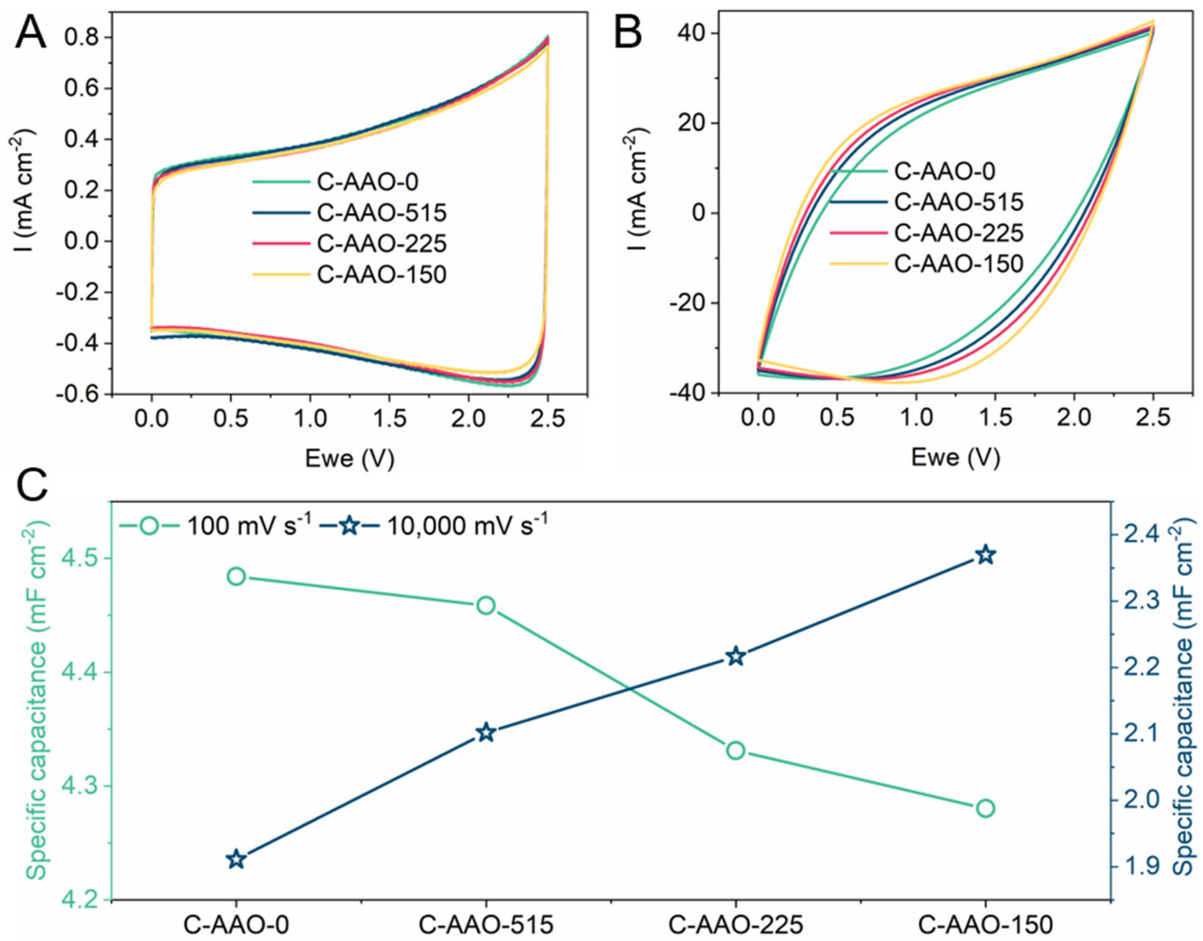

Figure S23. Cyclic voltammetry tests of C-AAO-0, C-AAO-515, C-AAO-225, and C-AAO-150 in pure Emim-BF_4 ionic liquid.

(A) CV curves at 100 mV s^{-1}.

(B) CV curves at 10,000 mV s^{-1}.

(C) Specific capacitance of C-AAO at 100 mV s^{-1} and 10,000 mV s^{-1}.

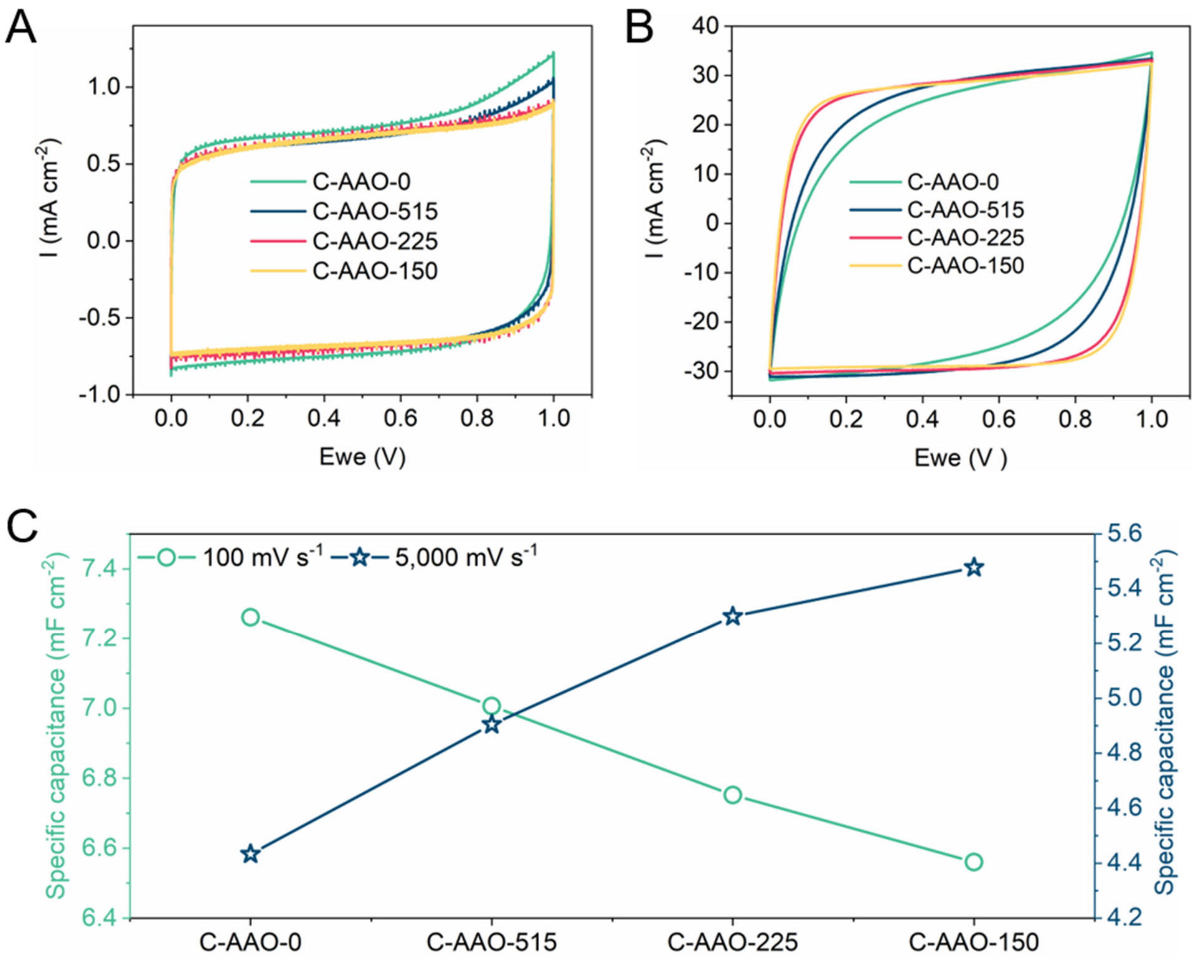

Figure S24. Cyclic voltammetry tests of C-AAO-0, C-AAO-515, C-AAO-225, and C-AAO-150 in 0.5 M Na_2SO_4 aqueous solution.

(A) CV curves at 100 mV s^{-1}.

(B) CV curves at 5,000 mV s^{-1}.

(C) Specific capacitance of C-AAO at 100 mV s^{-1} and 5,000 mV s^{-1}.

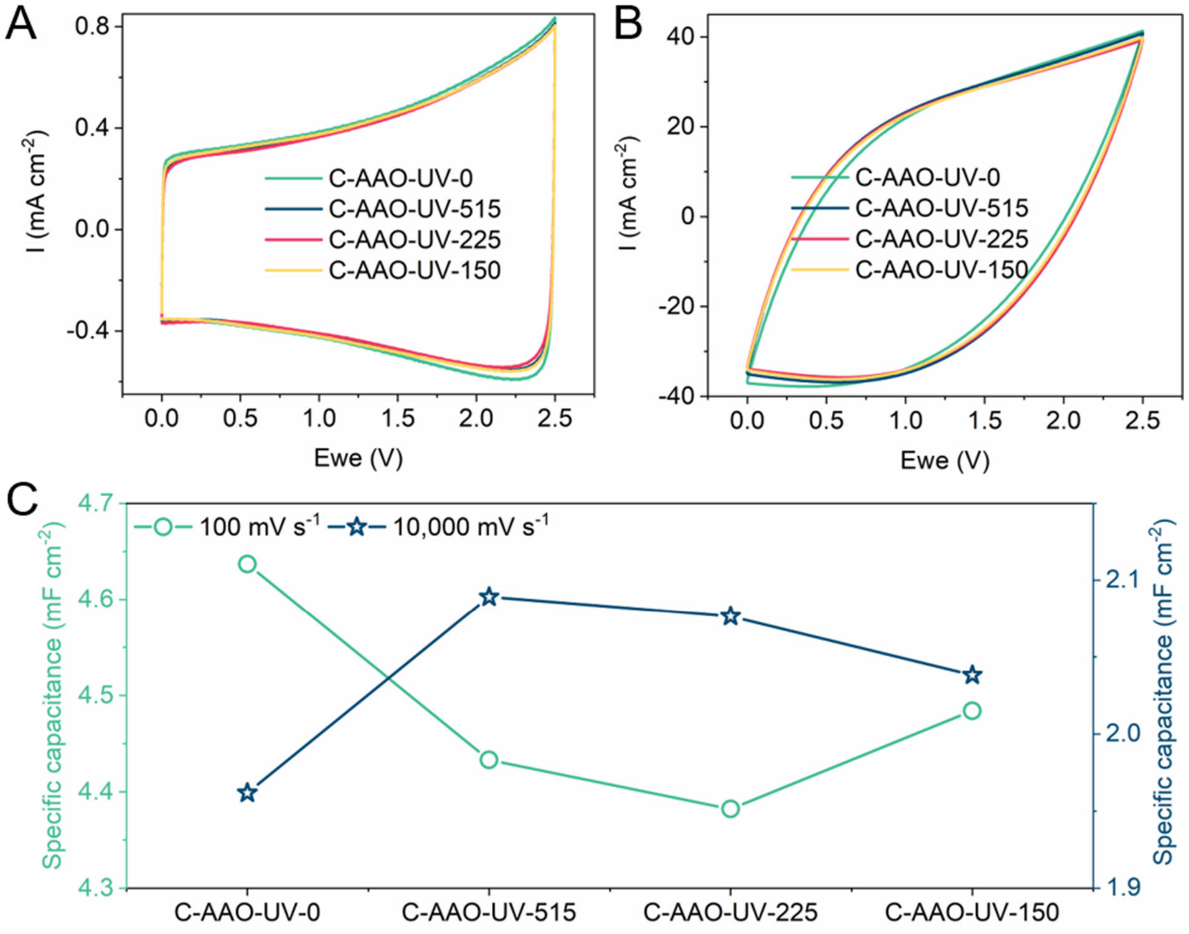

Figure S25. Cyclic voltammetry tests of C-AAO-UV-0, C-AAO-UV -515, C-AAO-UV -225, and C-AAO-UV-150 in pure Emim-BF$_4$ ionic liquid.

(A) CV curves at 100 mV s^{-1}.

(B) CV curves at 10,000 mV s^{-1}.

(C) Specific capacitance of C-AAO-UV at 100 mV s^{-1} and 10,000 mV s^{-1}.

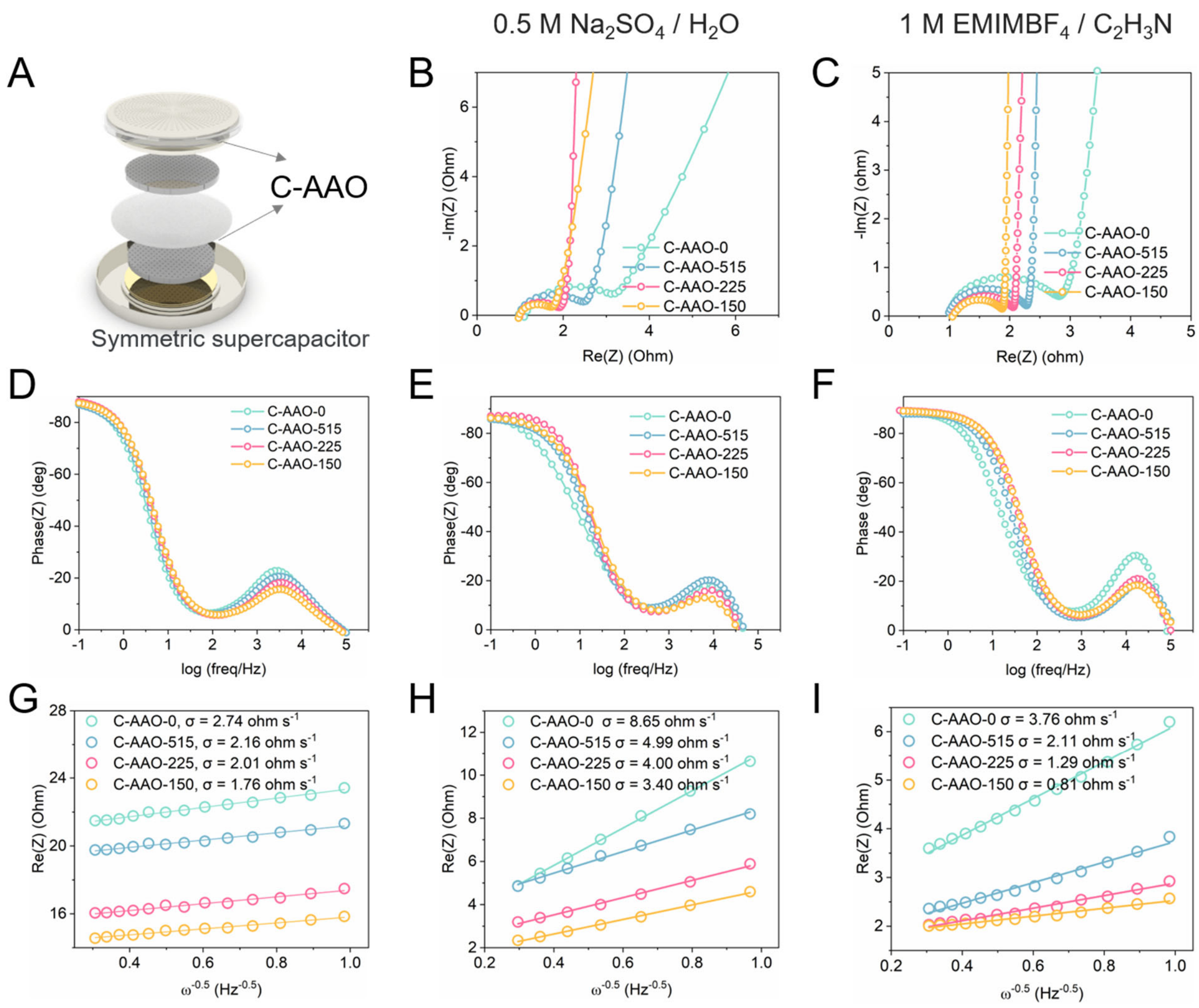

Figure S26. Impedance analysis of 3D C-AAO.

(A) Schematic diagram of the symmetrical supercapacitor used in the electrochemical test.

(B) Nyquist plot of 3D C-AAO in 0.5 M Na_2SO_4 aqueous solution.

(C) Nyquist plot of 3D C-AAO in 1 M Emim- BF_4 in acetonitrile solution.

(D) Phase angle versus frequency of 3D C-AAO in pure Emim-BF_4 ionic liquid.

(E) Phase angle versus frequency of 3D C-AAO in 0.5 M Na_2SO_4 aqueous solution.

(F) Phase angle versus frequency of 3D C-AAO in 1 M Emim-BF_4 in acetonitrile solution.

(G) Warburg coefficient (σ) of 3D C-AAO in pure Emim-BF_4 ionic liquid.

(H) Warburg coefficient (σ) of 3D C-AAO in 0.5 M Na_2SO_4 aqueous solution.

(I) Warburg coefficient (σ) of 3D C-AAO in 1 M Emim- BF_4 in acetonitrile solution.

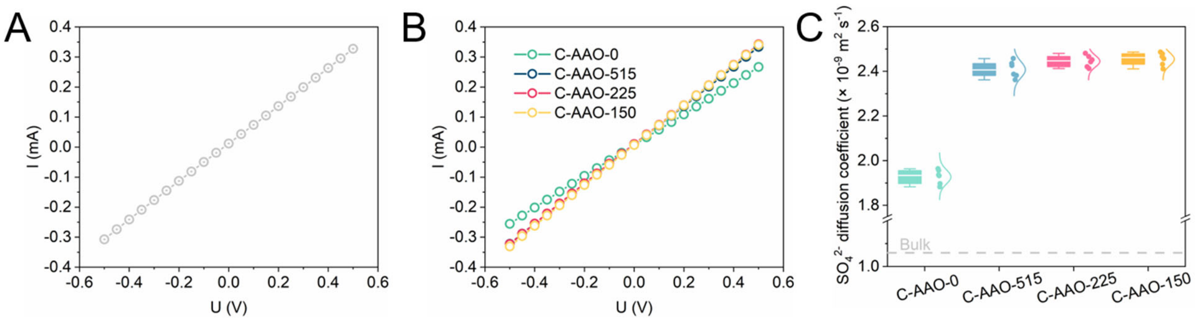

Figure S27. I-V scan.

(A) I-V curves without 3D C-AAO. In this control setup, the pore connecting the two electrochemical cells has a diameter of 0.2 cm.

(B) I-V curves of 3D C-AAO.

(C) SO_4^{2-} diffusion coefficient of 3D C-AAO electrodes, measured in 0.5 M Na_2SO_4 aqueous solution. The gray dashed line in the figure represents the SO_4^{2-} diffusion coefficient in the bulk solution.

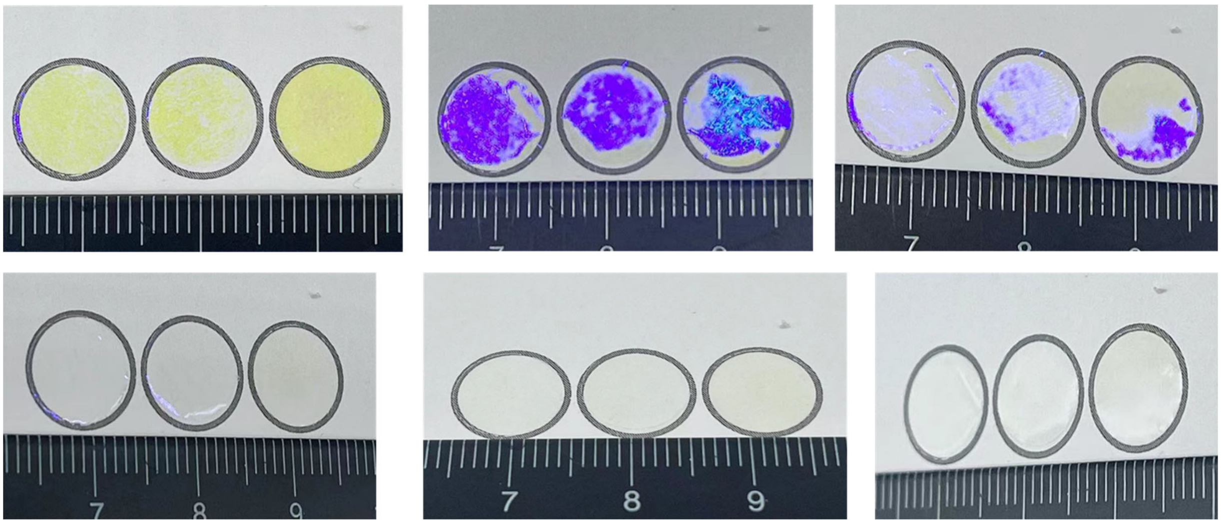

Figure S28. Optical images of 3D AAO precursors of C-AAO-T, C-AAO-M, and C-AAO-B from different angles.

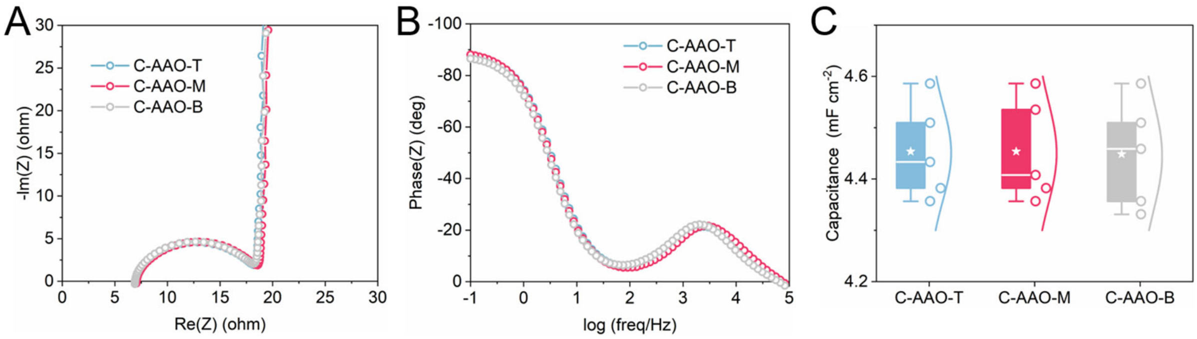

Figure S29. Electrochemical performance of C-AAO-T, C-AAO-M, and C-AAO-B.

(A) Nyquist plot.

(B) Phase angle versus frequency.

(C) Specific capacitance at 100 mV s^{-1}.

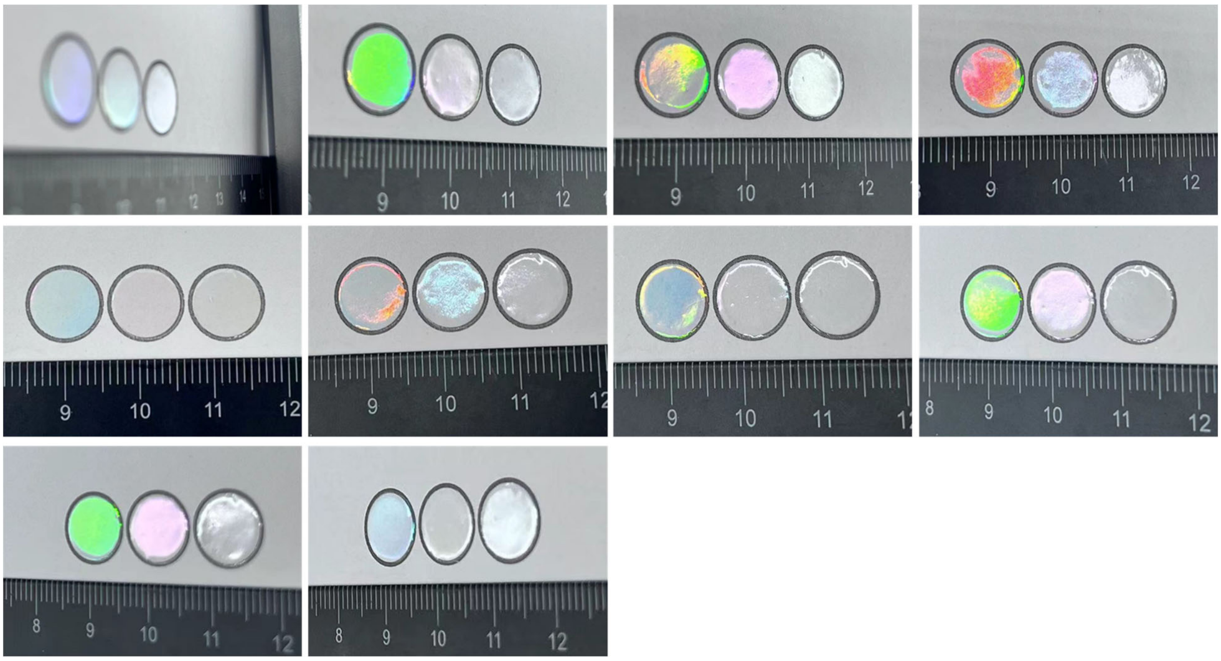

Figure S30. Optical images of 3D AAO precursors of C-AAO-U, C-AAO-G, and C-AAO-S from different angles.

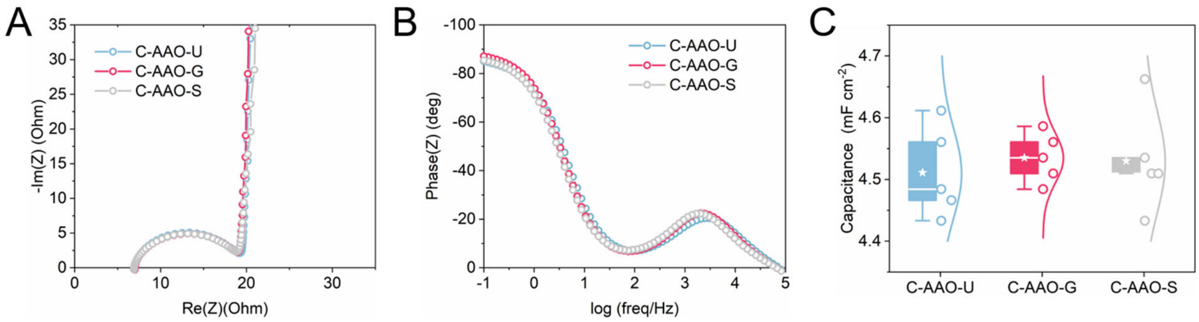

Figure S31. Electrochemical performance of C-AAO-U, C-AAO-G, and C-AAO-S.

(A) Nyquist plot.

(B) Phase angle versus frequency.

(C) Specific capacitance at 100 mV s^{-1}.

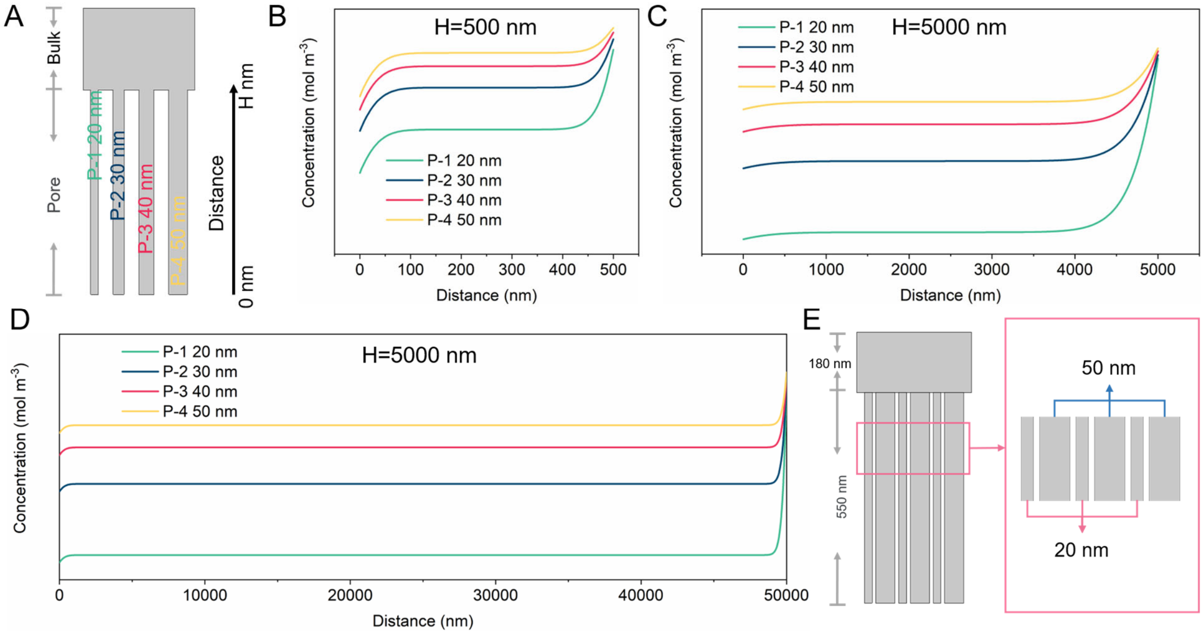

Figure S32. Ion concentration changes in pores during charging.

(A) Geometry of the model used to simulate ion concentration within pores. The model consists of a bulk electrolyte section and a porous electrode section. The bulk electrolyte section measures 300 nm × 0.4H nm. The porous electrode section has pore diameters are 20 nm, 30 nm, 40 nm, and 50 nm, with a pore length of H nm.

(B) Ion concentration within pores when H is 500 nm.

(C) Ion concentration within pores when H is 5000 nm.

(D) Ion concentration within pores when H is 50000 nm.

(E) Geometry of the model used in the main text for demonstration. This model consists of a bulk electrolyte section and a porous electrode section. The bulk electrolyte section measures 300 nm × 180 nm. The porous electrode section consists of alternating pores with dimensions of 20 nm × 550 nm and 50 nm × 550 nm.

Note of Figure S32: As shown in the figure, ion concentrations within individual nanopores of varying diameters differ throughout the charging process. Regardless of changes in pore length, the concentration gradient between pores of different sizes remains nearly constant along the entire length of the pore, from the opening to the bottom.

In our study, the C-AAO structures used typically exhibit pore sizes ranging from 40 to 50 nanometers. In the simulations, we initially planned to use values within this range (40 nm and 50 nm). The simulation results showed that while pore size differences influenced the ion concentration within individual pores during charging, they did not alter the overall distribution trend (Figure S32). To enhance the visual contrast of the simulation results, we set the aperture of the smaller pores to 20 nm and that of the larger pores to 50 nm.

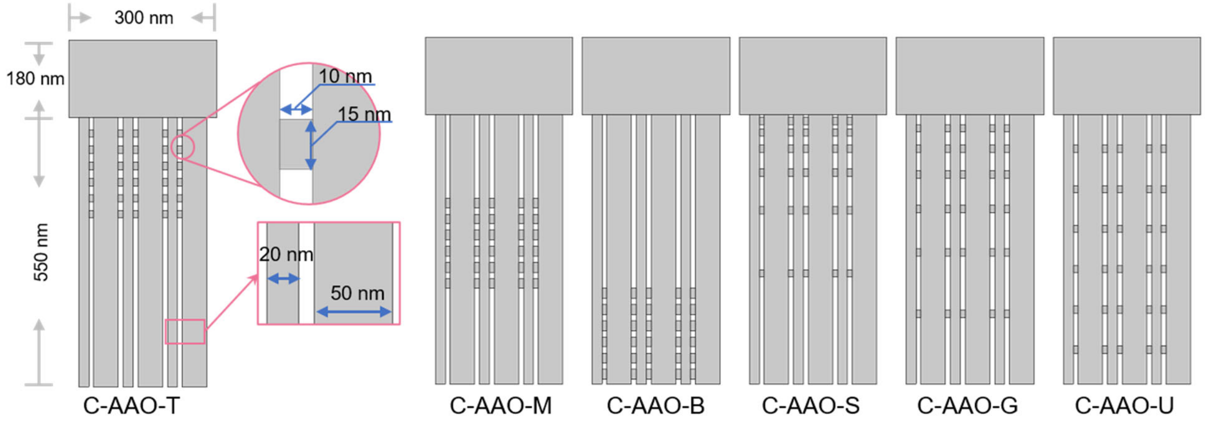

Figure S33. Geometry of the 3D C-AAO model used for simulation.

Note of Figure S33: The model consists of a bulk electrolyte section and a porous electrode section. The bulk electrolyte section measures 300 nm × 180 nm. The porous electrode section consists of straight and transversal pores, where the straight pores are 20 nm × 550 nm and 50 nm × 550 nm, and the transversal pores are 10 nm × 15 nm. For C-AAO-T, C-AAO-M, and C-AAO-(B) the spacing between transversal pores is 33 nm. For C-AAO-U, the transversal pore spacing is 82 nm. For C-AAO-G, the transversal pore spacing is 35 nm, 42 nm, 52 nm, 70 nm, 90 nm, and 125 nm, respectively. For C-AAO-S, the transversal pore spacing is 20 nm, 24 nm, 33 nm, 48 nm, 77 nm and 130 nm, respectively.

SUPPLEMENTAL TABLES

Table S1. Anodization Parameters for all C-AAO Samples

Sample	Pulsed Anodization (Number of Pulses)	Pulse Interval (s)	Constant Voltage Anodization (s)	Notes
C-AAO-0	None	None	22200	Constant Voltage only
C-AAO-515	36	600 (fixed)	None	Pulsed only
C-AAO-225	84	240 (fixed)	None	Pulsed only
C-AAO-150	124	150 (fixed)	None	Pulsed only
C-AAO-T	36	200 (fixed)	14000 (after pulses)	Pulsed-constant
C-AAO-M	36	200 (fixed)	7000 (before and after pulses)	Constant-Pulsed-Constant
C-AAO-B	36	200 (fixed)	14000 (before pulses)	Constant–Pulsed
C-AAO-U	36	600 (fixed)	None	Pulsed only
C-AAO-G	36	405 (+11 increment)	None	First pulse 405 s, each subsequent pulse increases by 11 s, last pulse 790 s
C-AAO-S	36	160 (+25 increment)	None	First pulse 160 s, each subsequent pulse increases by 25 s, last pulse 1035 s

Note of Table S4: The limiting current during the pulse process is 20 mA cm^{-2}, and the pulse time is uniformly 1 s.

Table S2. Variation of pore size and transversal pores in 3D C-AAO samples with different qualities

C-AAO-0		
Weight (mg, ± 0.02 mg)	Aperture (nm, ± 5.0 nm)	Pore structure
1.86	43.1	Standard vertical circular pore
1.81	44.1	Standard vertical circular pore
1.69	45.6	Standard vertical circular pore
1.60	46.4	Standard vertical circular pore
1.55	46.8	Standard vertical circular pore
1.45	47.5	Standard vertical circular pore
1.40	47.8	Standard vertical circular pore
C-AAO-515		
Weight (mg, ± 0.02 mg)	Aperture (nm, ± 5.0 nm)	Pore structure
1.70	44.0	Bamboo-like
1.61	45.0	Bamboo-like
1.51	45.9	Transversal
1.48	46.1	Transversal
1.35	46.9	Transversal
1.30	47.2	Transversal
1.22	47.6	Transversal
1.12	48.0	Transversal
C-AAO-225		
Weight (mg, ± 0.02 mg)	Aperture (nm, ± 5.0 nm)	Pore structure
1.61	43.8	Bamboo-like
1.51	44.8	Bamboo-like
1.39	45.8	Transversal
1.29	46.4	Transversal
1.24	46.7	Transversal
1.19	46.9	Transversal
1.10	47.3	Transversal
0.96	47.7	Transversal
C-AAO-150		
Weight (mg, ± 0.02 mg)	Aperture (nm, ± 5.0 nm)	Pore structure
1.51	43.7	Bamboo-like
1.46	44.2	Bamboo-like
1.39	44.9	Bamboo-like
1.30	45.5	Bamboo-like
1.17	46.2	Transversal
1.12	46.4	Transversal

1.03	46.7	Transversal

Table S3. Volume, surface area and specific capacitance changes of 3D C-AAO samples with the same pore size

Sample	$V_{3D\text{-}C\text{-}AAO}/V_{C\text{-}AAO\text{-}0}$	$S_{3D\text{-}C\text{-}AAO}/S_{C\text{-}AAO}$	$C_{3D\text{-}C\text{-}AAO}/C_{C\text{-}AAO}$
C-AAO-0	1	1	1
C-AAO-515	1.04	0.98	0.99
C-AAO-225	1.10	0.95	0.96
C-AAO-150	1.15	0.93	0.95

Table S4. Variations of pore size, surface area and specific capacitance of 3D C-AAO samples with the same pore volume

Sample	Aperture (nm)	$S_{3D\text{-}C\text{-}AAO\text{-}UV}/S_{C\text{-}AAO\text{-}0\text{-}UV}$	$C_{3D\text{-}C\text{-}AAO\text{-}UV}/C_{C\text{-}AAO\text{-}0\text{-}UV}$
C-AAO-0-UV	~ 47.0	1	1
C-AAO-515-UV	~ 46.0	0.96	0.96
C-AAO-225-UV	~ 44.8	0.95	0.95
C-AAO-150-UV	~ 43.7	0.99	0.97
